# Infant and young child feeding practices in Lebanon: a cross-sectional national study

**DOI:** 10.1017/S1368980022000842

**Published:** 2022-04-04

**Authors:** Farah Naja, Nahla Hwalla, Fatima Al Zahraa Chokor, Rasha Zgheib, Lara Nasreddine

**Affiliations:** 1 Department of Nutrition and Food Sciences, American University of Beirut, P.O. Box 11-0236, Riad El-Solh, Beirut 1107 2020, Lebanon; 2 Department of Clinical Nutrition and Dietetics, Research Institute of Medical & Health Sciences (RIMHS), College of Health Sciences, University of Sharjah, Sharjah, United Arab Emirates; 3 Inserm 1256 NGERE, Nancy, France

**Keywords:** First 1000 days, Breast-feeding practices, Complementary feeding practices, Determinants, Lebanon

## Abstract

**Objective:**

To assess infant and young child feeding (IYCF) practices in Lebanon and investigate their associations with socio-demographic and lifestyle factors.

**Design:**

A cross-sectional national survey was conducted in 2012–2013. In addition to a socio-demographic and lifestyle questionnaire, a 24-h dietary recall for the children was collected, with mothers as proxies. IYCF practices were assessed based on the 2021 indicators of the WHO.

**Setting:**

Lebanon.

**Participants:**

Children aged 0–23 months and their mothers (*n* 469).

**Results:**

While the majority of infants were ever breastfed (87·6 %), the prevalence of exclusive breast-feeding (BF) in those under 6 months of age was 11·0 %. Early initiation of BF was 28 %. A greater child’s birth order, partner’s support for BF, higher parental education, maternal BF knowledge and non-smoking were associated with higher odds of meeting BF recommendations. As for complementary feeding, 92·8 % of children (6–23 months) met the minimum meal frequency indicator, 37·5 % met the minimum dietary diversity (MDD) and 34·4 % met the minimum adequate diet (MAD). The consumption of unhealthy food was observed amongst 48·9 % of children, with nearly 37 % consuming sweet beverages. Older maternal age and maternal overweight/obesity were associated with lower odds of meeting MDD and MAD, while child’s age and partner’s support for BF were associated with higher odds.

**Conclusions:**

The results documented suboptimal IYCF practices amongst Lebanese children and identified a number of factors associated with these practices. Findings from this study will help guide the development of culture-specific programmes aimed at improving IYCF practices in Lebanon.

The first ‘1000 days’ of life, which cover the period between conception and the child’s second birthday, is characterised by high plasticity and rapid development of organs and systems, including the brain, immune and neural systems as well as the gut microbiome^(1)^. As such, this period of the lifecycle represents a unique window of opportunity to shape growth and lay the foundations for optimal health and development^(2)^. The WHO endorsed exclusive breast-feeding (EBF) for the first 6 months of life, and the safe introduction of complementary foods thereafter, with continued breast-feeding (BF) up to 2 years of age^(3)^. These adequate infant and young child feeding (IYCF) practices not only affect growth and development of infants’ vital organs but also play a critical role in programing health outcomes such as adult onset non-communicable diseases, morbidity risk and the quality of life in adulthood^(2)^.

Despite these recommendations and the well-established benefits of adequate IYCF practices, feeding patterns remained suboptimal in many countries around the world, including those of the Eastern Mediterranean Region (EMR). For instance, the proportion of mothers who reported EBF for 6 months was estimated at 29·3 % in the region^(4)^, while a common observation to all countries of the EMR was the practice of mixed BF and bottle-feeding as early as the first month of life, as well as the premature introduction of complementary foods^(5)^. In Lebanon, a small country of the Eastern Mediterranean basin, the prevalence of EBF for 6 months was estimated at 10·1 % in a national sample of mother–child pairs recruited in 2004 from primary healthcare centres^(6)^, and complementary feeding (CF) practices were suggested to be inadequate^(7)^. In the majority of available studies in Lebanon, IYCF practices were assessed based on a retrospective recall, which may potentially involve a recall bias, particularly among mothers of older children^(8–10)^. To enhance IYCF assessment, foster programmatic action and contribute to progress monitoring, the WHO and the United Nations Children’s Fund (UNICEF) recommended assessing IYCF based on the current status of feeding among infants and young children less than 2 years of age, that is, the current age of the child and a recall of what the child has consumed in terms of food and beverages during the past 24 h^(11)^.

Besides the assessment and characterisation of context-specific patterns of IYCF, it is also crucial to understand the determinants of IYCF practices in a given population, as an essential prerequisite for the development of effective promotion strategies and interventions^(12)^. Research investigating the factors associated with inadequate BF and/or CF practices has suggested an association with certain demographic, socio-economic or lifestyle-related factors. For instance, delayed initiation of BF and absence of EBF during the first 6 months were affected by factors such as maternal age^(13)^, maternal education and employment^(13)^, maternal nutritional status^(8)^, mode of delivery^(8,13)^, as well as household wealth status^(8)^. Studies investigating complementary practices have also shown that younger maternal age^(14)^, lower maternal education^(14,15)^, maternal unemployment^(14,16)^, young infant age^(15,16)^ and poor socio-economic status^(15,16)^ were amongst the main factors associated with inappropriate CF practices. Knowing that early nutrition is a crucial aspect of young child’s health, this study aimed to characterise feeding practices among 0–2-year-old Lebanese children, using data from the national survey conducted in 2012–2013. More specifically, the objectives of this study were to (1) assess IYCF practices in Lebanon, as per the recent 2021 WHO/UNICEF IYCF guidelines and (2) identify the demographic, socio-economic and lifestyle factors that are associated with inappropriate IYCF practices in Lebanon. Findings of this study may be used for the development of evidence-based and culture-specific recommendations and interventions aimed at optimising IYCF in Lebanon.

## Materials and methods

### Study design

This cross-sectional study was based on data collected as part of the national survey entitled ‘*Early Life Nutrition and Health in Lebanon’* (ELNAHL). The survey was conducted over a year from September 2012 to August 2013, on a representative sample of 0–5-year-old children and their mothers. Details pertinent to this survey are published elsewhere^(17)^. The primary sampling unit was the household. The selection of households was conducted based on a stratified cluster sampling strategy, with the strata being the six governorates of Lebanon and the clusters selected further at the level of districts. In each district, the selection of households was performed based on a probability proportional to size approach. Within the districts, the selection of households was carried out using systematic sampling^(17)^.

Sample size calculation for the ELNAHL survey was based on an estimated prevalence of 13 % of overweight and obesity amongst under-five children (U5)^(18)^. Accordingly, a sample of 1030 under-five children was needed to estimate the prevalence of overweight and obesity with a 2 % error and a 95 % CI. To be eligible for participation in the survey, households had to include a mother (18 years of age or older) and a child aged 5 years or below. Of the 1194 eligible households who were contacted, 1029 participated in the survey, with a response rate of 86 %. This recruited sample constituted 99·9 % of the estimated sample (1029/1030 × 100). Exclusion criteria included having a non-Lebanese nationality, being born preterm (<37 weeks) or suffering from any chronic illness, inborn errors of metabolism or physical malformations that may alter dietary intake. For the present study, data related to children aged 0–23 months and their mothers were included (*n* 469). ELNAHL survey was carried out as per the guidelines specified by the Declaration of Helsinki, and the study protocol and procedures were reviewed and approved by the Institutional Research Board, American University of Beirut (Protocol number NUT.LN.13). All participants provided a written consent form before participating in the study.

### Data collection

Data were obtained in the household through face-to-face interviews with the mother. Each interview lasted approximately 1 h. Nutritionists collected data using a multicomponent questionnaire. Before conducting fieldwork, the nutritionists were extensively trained on the data collection protocols, including the dietary and anthropometric assessments. In addition, they were coached on how to maintain a welcoming yet non-judgmental attitude towards participants. The questionnaire was pilot-tested on a sample of fifteen mothers, prior to its adoption in the survey. In addition to the questionnaire, anthropometric measurements (weight and height) of the mother were obtained, and her BMI was calculated.

### Characteristics of study participants

The characteristics of study participants were categorised as individual, household and community-level factors, as per the conceptualisation published by Victor *et al.* ^(13,16)^. The individual-level section included age of mother and child, sex of the child, education level and employment status of mother and partner, child’s birth order, mode of delivery, maternal BMI, maternal smoking (ever *v*. never) and alcohol drinking (ever *v*. never), as well as her BF knowledge. The latter was examined using the Arabic Breastfeeding Knowledge Questionnaire (BFK-A), an adaptation of the original Infant Feeding Knowledge Test Form (AFORM) originally developed by Grossman *et al.* ^(19,20)^. Household-level characteristics included questions related to income, presence of paid helper and whether the partner provided positive support for BF. The last section was dedicated to community-level characteristics, with questions related to governorate of residence and whether the hospital provided any support for BF after delivery.

### Assessment of infant and young child feeding practices

During the household visit, a 24-h dietary recall was administered to mothers participating in the survey. The 24-h dietary recall inquired about all foods and beverages consumed by the child during the 24-h period preceding the interview. During data collection, specific attention was given to foods consumed at daycare, if applicable. In case another caretaker shared the responsibility of feeding the child, the mother was asked to consult with the caretaker for further information related to the dietary interview.

IYCF practices were assessed using the recent WHO/UNICEF IYCF guidelines. The definition and calculation method for each of the IYCF indicators is included in online supplementary material, Supplemental Table 1. Based on the 24-h recall, the following BF indicators were assessed: EBF under 6 months (among infants 0–5 months of age); mixed milk feeding under 6 months (among 0–5 months of age) and continued BF (among 12–23-month-old children). The indicators on ever breastfed (among 0–23-month-old-children), early BF initiation (within 1 h of birth; among 0–23-month-old children) and EBF for the first 2 d after birth (among 0–23-month-old children) were assessed based on specific questions in the survey questionnaire (Was your child ever breastfed?; How long did you wait after birth before placing your child to the breast? After delivery and during your stay in hospital did your infant receive any fluids or formula milk within the first 2 d? (yes or no).)

The evaluated CF indicators included the following: introduction of solid, semi-solid or soft foods (among 6–8-month-old children); minimum dietary diversity (MDD; among 6–23-month-old children); minimum meal frequency (MMF; among 6–23-month- old children); minimum milk feeding frequency for non-breastfed children (among non-breastfed 6–23-month-old children); minimum acceptable diet (MAD; among 6–23-month-old children); egg and/or flesh food consumption (among 6–23-month-old children); sweet beverage consumption (among 6–23-month-old children); unhealthy food consumption (among 6–23-month-old children) and no vegetable or fruit consumption (among 6–23-month-old children).

Except for the indicators ‘ever breastfed’, ‘early initiation of breast-feeding’ and ‘exclusively breastfed for the first two days after birth’, the evaluation of all other indicators was based on current status data, that is, the current age of the child and information pertinent to the day preceding the interview.

### Anthropometric assessment

Anthropometric measurements were obtained for the mother, including weight (kg) and height (m), which were measured according to standard protocols^(21)^, using a scale (SECA 770) and a portable stadiometer (SECA 213), respectively. Maternal BMI was calculated as weight (kg)/height (m^2^), and mothers were classified as underweight (BMI < 18·5 kg/m^2^), normal weight (BMI 18·5 to <25 kg/m^2^), overweight (BMI 25 to <30 kg/m^2^) or obese (BMI ≥ 30 kg/m^2^)^(22)^.

### Data analysis

Subject characteristics, including IYCF practices, were described as frequency and proportions for categorical variables and mean ± se for continuous variables. In the analysis, missing data were replaced by the mode. The proportion of missing responses in the data ranged from 0·2 to 6·6 %, except for income where 21 % of entries were missing. Therefore, for the variable ‘income’, a separate category ‘does not know/refused to answer’ was created and added as a fourth level. Various validity checks were carried out before analysis, including data type, range and consistency checks.

In order to examine the association of the individual, household and community characteristics with the IYCF indicators, simple and multiple regressions analyses were conducted. The block logistic regression was used in the modelling as it allowed to derive a multilevel model which corresponded to the various levels of subjects’ characteristics (individual, household and community). In these analyses, the IYCF indicators were dependent variables, expressed as yes or no. All analyses were weighted by sample weights using ‘svy’ commands to account for the stratified two-stage cluster sampling design and were conducted using Stata (StataCorp. 2019. Stata: Release 16. Statistical Software. StataCorp., LLC). Unadjusted OR with 95 % CI were calculated, and variables with *P* < 0·2 and/or variables which are important according to the literature were retained in the multiple logistic regression. A *P*-value less than 0·05 was considered statistically significant.

## Results

Table 1 describes the participants’ characteristics, categorised as individual, household or community-level characteristics. The mean age of participating mothers was 29·98 ± 6·33 years, and the majority (99·8 %) were married. For both mothers and partners, over 67 % had an intermediate, high-school or technical diploma education level, but a higher percentage of mothers reported a high-school degree compared with partners (23·7 % *v*. 15·1 %). Most of the participating mothers (80·2 %) were unemployed at the time of the interview, while 95·9 % of partners were reported as working. The sex distribution of participating children was 49 % for boys and 51 % for girls, and their age distribution was as follows: 21·7 % younger than 6 months (mean ± sd: 2·8 ± 1·8 months), 31·6 % between 6 and 11 months (mean ± sd: 9·0 ± 1·7 months) and 46·7 % between 12 and 23 months (mean ± sd: 18·4 ± 3·6 months). The proportions of women who reported a Caesarean section delivery was 45 %, compared with 55 % reporting vaginal delivery. Regarding their lifestyle characteristics, the prevalence of smoking (including past smokers) was 39·2 % and that of ever alcohol drinking at 15·1 %. Only 31·3 % of the mothers reported a high level of BF knowledge, while 23·2 % were classified as obese.

**Table 1:** Individual, household and community-level characteristics of study participants (children 0–23 months of age and their mothers), Lebanon 2012–2013 (*n* 469)

	*n*	%
Individual-level characteristics		
Maternal age (years)		
15–24	97	20·7
25–34	255	54·4
35–49	117	24·9
Maternal education level		
Primary school or less	44	9·4
Intermediate, high school or technical diploma	314	67·0
University degree or higher	111	23·7
Partner’s education level		
Primary school or less	83	17·7
Intermediate, high school or technical diploma	315	67·2
University degree or higher	71	15·1
Maternal working status		
Working	93	19·8
Not working	376	80·2
Partner’s working status		
Working	450	95·9
Not working	19	4·1
Sex of the child		
Male	230	49·0
Female	239	51·0
Child’s age (months)		
0–5	102	21·7
6–11	148	31·6
12–23	219	46·7
Birth order		
First born	184	39·2
Second to fourth	248	52·9
Fifth or more	37	7·9
Mode of delivery		
Vaginal delivery	258	55·0
Caesarean section delivery	211	45·0
Maternal BMI		
Underweight	5	1·1
Normal	181	38·6
Overweight	174	37·1
Obese	109	23·2
Maternal smoking		
Ever	184	39·2
Never	285	60·8
Alcohol drinking		
Ever	71	15·1
Never	398	84·9
Tertiles of maternal knowledge score related to breast-feeding		
1 (lowest)	219	46·7
2	103	22·0
3 (highest)	147	31·3
Household-level characteristics		
Household monthly income (Lebanese Pounds)*		
<1 000 000	166	35·4
1 000 000–2 000 000	133	28·4
>2 000 000	73	15·6
Doesn’t know/refused to answer	97	20·7
Presence of paid helper in the household		
No	388	82·7
Yes	81	17·3
Partner providing positive support for breast-feeding		
No (including neutral)	53	11·3
Yes	416	88·7
Community-level characteristics		
Governorate		
Beirut	37	7·9
Mount Lebanon	138	29·4
North	186	39·7
South and Nabatiyeh	71	15·1
Bekaa	37	7·9
Hospital providing support for breast-feeding your child after delivery		
Yes	386	82·3
No/neutral	83	17·7

* 1 USD = 1500 Lebanese Pounds at the time of the survey.

For the majority of participants (63·8 %), household income was 2 Million Lebanese Pounds or lower (approximately 1333 USD at the time of the survey), while 82·7 % of participants reported having no helper at home. The majority of partners were supportive of BF (88·7 %). As for the community-level factors, the North governorate as well as Mount Lebanon had the highest proportions of participants (39·7 and 29·4 %, respectively), and the majority of mothers (82·3 %) reported giving birth in hospitals that supported BF (Table 1).

BF and CF indicators are displayed in Table 2. Ever BF was reported among 87·6 % of participants, while the prevalence of early BF initiation and EBF for the first 2 d after birth was only 28 and 28·6 %, respectively. The prevalence of EBF and mixed milk feeding amongst children under 6 months of age was 11 and 42·1 %, respectively. All children who received mixed milk feeding were given formula in addition to breast milk, and none was fed animal milk. The proportion of 12–23 months aged children who continued to breastfeed was 13·6 %.

**Table 2:** Prevalence of breast-feeding and complementary feeding practice indicators among children 0–23 months of age, Lebanon 2012–2013

	*n*	%	95 % CI§§§
Breast-feeding indicators			
Ever breastfed*			
Yes	413	87·6	82·9, 91·1
No	56	12·4	8·9, 17·0
Early initiation of breast-feeding†			
Yes	121	28·0	20·1, 37·5
No	348	72·0	62·5, 79·9
Exclusively breastfed for the first 2 d after birth‡			
Yes	142	28·6	22·6, 35·4
No	327	71·4	64·5, 77·4
Exclusive breast-feeding under 6 months§ (among 0–5 months)			
Yes	20	11·0	5·2, 21·9
No	82	89·0	78·1, 94·8
Mixed milk feeding under 6 months‖ (among 0–5 months)			
Yes	42	42·1	22·1, 65·1
No	60	57·9	34·9, 77·9
Continued breast-feeding 12–23 months¶ (among 12–23 months)			
Yes	23	13·6	7·9, 22·5
No	196	86·4	77·5, 92·1
Complementary feeding indicators			
Introduction of solid, semi-solid or soft foods 6–8 months**			
Yes	69	96·2	89·9, 98·6
No	5	3·8	1·4, 10·1
Minimum dietary diversity††			
Yes	137	37·5	30·5, 45·0
No	230	62·5	55·0, 69·4
Minimum meal frequency (MMF)‡‡			
Yes	346	92·8	87·9, 95·8
No	21	7·2	4·2, 12·1
MMF for breastfed children‡‡			
Yes	68	81·3	66·0, 90·7
No	12	18·7	9·3, 34·0
MMF for non-breastfed children‡‡			
Yes	278	96·5	91·8, 98·5
No	9	3·5	1·5, 8·2
Minimum milk feeding frequency for non-breastfed children 6–23 months§§			
Yes	258	90·0	83·7, 94·0
No	29	10·0	6·0, 16·3
Minimum acceptable diet (MAD)‖‖			
Yes	125	34·4	27·8, 41·7
No	242	65·6	58·3, 72·2
MAD for breastfed children‖‖			
Yes	42	49·4	35·0, 63·9
No	38	50·6	36·1, 65·0
MAD for non-breastfed children‖‖			
Yes	83	29·7	23·1, 37·3
No	204	70·3	62·7, 76·9
Egg and/or flesh food consumption 6–23 months¶¶			
Yes	185	48·0	40·2, 55·9
No	182	52·0	44·1, 59·8
Sweet beverage consumption 6–23 months***			
Yes	143	36·9	31·2, 42·9
No	224	63·1	57·1, 68·8
Unhealthy food consumption 6–23 months†††			
Yes	169	48·9	39·9, 57·9
No	198	51·1	42·1, 60·1
No vegetable or fruit consumption 6–23 months‡‡‡			
Yes	76	21·3	15·3, 28·9
No	291	78·7	71·2, 84·7

* Proportion of children born in the last 24 months who were ever breastfed (questionnaire) (*n* 469).

† Proportion of children born in the last 24 months who were put to the breast within 1 h of birth (questionnaire) (*n* 469).

‡ Proportion of children born in the last 24 months who were fed exclusively with breast milk for the first 2 d after birth (questionnaire) (*n* 469).

§ Proportion of infants 0–5 months of age who are fed exclusively with breast milk during the previous day (*n* 102).

‖ Proportion of infants 0–5 months of age who were fed formula and/or animal milk in addition to breast milk during the previous day (*n* 102).

¶ Proportion of children 12–23 months of age who were fed breast milk during the previous day (*n* 219).

** Proportion of infants 6–8 months of age who receive solid, semi-solid or soft foods during the previous day (*n* 74).

†† Proportion of children 6–23 months of age who consumed foods and beverages from at least five out of eight defined food groups during the previous day (*n* 367).

‡‡ Proportion of breastfed and non-breastfed children 6–23 months of age who consumed solid, semi-solid or soft foods (including milk feeds for non-breastfed children) the minimum number of times or more during the previous day (*n* 367, 80 breastfed and 287 non-breastfed children).

§§ Proportion of non-breastfed children 6–23 months of age who consumed at least two milk feeds during the previous day (*n* 287).

‖‖ Proportion of children 6–23 months of age who consumed a minimum acceptable diet during the previous day (*n* 367, 80 breastfed and 287 non-breastfed children).

¶¶ Proportion of children 6–23 months of age who consumed egg and/or flesh food during the previous day (*n* 367).

*** Proportion of children 6–23 months of age who consumed a sweet beverage during the previous day (*n* 367).

††† Proportion of children 6–23 months of age who consumed selected sentinel unhealthy foods during the previous day (*n* 367).

‡‡‡ Proportion of children 6–23 months of age who did not consume any vegetables or fruits during the previous day (*n* 367).

§§§ Percentages presented in the table are weighted percentages.

The introduction of solid, semi-solid or soft food at 6–8 months of age was met by 96·2 % of the study sample. In children aged 6–23 months, 37·5, 92·8 and 34·4 % were meeting the MDD, MMF and MAD indicators, respectively (Table 2). The consumption of unhealthy food was observed in almost half of the children (48·9 %) aged 6–23 months, with nearly 37 % consuming a sweet beverage on the day prior to the interview. Approximately one-fifth of children (21·3 %) aged 6–23 months had no vegetable or fruit consumption during the previous day. Further description of the consumption of the various food groups within the MDD is presented in Table 3. The proportion of children meeting the MDD indicator was the lowest (24·9 %) in the youngest age group (6–11 months), while the highest proportion (46·5 %) was observed in older children aged 8–23 months. Amongst children aged 6–11 months, 93·7 % had reported the consumption of grains, roots and tubers, and 85·7 % had consumed fruits and vegetables. On the other hand, flesh foods and eggs were consumed by 29·9 and 3·6 % of children in this age group, respectively. In older children, the frequency of consumption of both of these two groups, flesh foods and eggs, increased (12–17 months: 51·9 and 22·2 %; 18–23 months: 55·7 and 20·1 %, respectively) (Table 3).

**Table 3:** Consumption of food groups by child age in months, Lebanon 2012–2013*

Child’s age in months	Meeting MDD		Breast milk		Grains, roots and tubers		Pulses		Dairy products		Flesh foods		Eggs		Vitamin A rich fruits and vegetables		Other fruits and vegetables	
	%	95 % CI	%	95 % CI	%	95 % CI	%	95 % CI	%	95 % CI	%	95 % CI	%	95 % CI	%	95 % CI	%	95 % CI
6–11 (*n* 148)	24·9	16·9, 35·1	39·3	26·8, 53·4	93·7	87·1, 97·0	22·5	13·8, 34·5	88·8	78·9, 94·4	29·9	19·1, 43·4	3·6	1·1, 11·3	20·9	13·2, 31·6	64·8	53·2, 74·8
12–17 (*n* 96)	45·2	32·8, 58·3	16·3	8·2, 29·6	95·9	81·6, 99·2	37·9	27·1, 50·0	91·9	77·4, 97·4	51·9	40·2, 63·3	22·2	11·6, 38·2	27	16·4, 41·2	83·2	71·8, 90·6
18–23 (*n* 123)	46·5	34·7, 58·7	11·9	5·5, 23·8	94·2	84·0, 98·0	35·7	24·9, 48·2	94·7	83·5, 98·4	55·7	44·2, 66·5	20·1	11·2, 33·3	38·2	23·5, 55·5	88·5	79·0, 94·0
6–23 (*n* 367)	37·5	30·5, 45·0	24	16·8, 32·9	94·4	90·6, 96·7	30·9	25·0, 37·5	91·6	84·9, 95·5	44·4	36·3, 52·8	14·0	9·1, 20·9	28·6	21·7, 36·5	77·7	70·2, 83·7

MDD, minimum diet diversity.

* Percentages presented in the table are weighted percentages.

The results of the simple logistic regression models describing the factors associated with various IYCF indicators are presented in online supplementary material, Supplemental Tables 2, 3a, and 3b.

The results of the block logistic regression models for BF indicators are presented in Table 4, and the corresponding models’ statistics are shown in online supplementary material, Supplemental Table 4. Amongst the individual-level variables, higher birth order and greater maternal BF knowledge were associated with higher odds of ever BF; higher maternal and paternal educational levels were associated with higher odds of early initiation of BF; a higher birth order and greater maternal BF knowledge were directly associated with EBF for the first 2 d, while obesity in mothers was associated with lower odds (adjusted OR (AOR) = 0·27, *P* = 0·018); non-smokers and mothers with higher BF knowledge were more likely to exclusively BF for up to 6 months (AOR = 9·82 with *P* = 0·038 and AOR = 9·09 with *P* = 0·017, respectively) while older mothers were less likely to do so (AOR = 0·07, *P* = 0·028); lastly, maternal non-smoking was associated with lower odds of mixed milk feeding under 6 months of age (AOR = 0·21, *P* = 0·028), while a higher maternal BF knowledge was associated with higher odds of continued BF at 12–23 months (AOR = 7·09, *P* = 0·002) (Table 4). The blocks of individual-level variables were significant (*P* < 0·05) in predicting all BF indicators except for mixed milk feeding and EBF under 6 months (online supplementary material, Supplemental Table 4).

**Table 4:** Adjusted odds ratios (AOR) and their corresponding 95 % CI for the association of various characteristics with different breast-feeding indicators among children, Lebanon 2012–2013*

Variables	Ever breastfed (*n* 469)		Early initiation of breast-feeding (*n* 469)		Exclusively breastfed for the first 2 d (*n* 469)		Exclusive breast-feeding under 6 months (*n* 102) (among 0–5·9 months)		Mixed milk feeding under 6 months (*n* 102) (among 0–5 months)		Continued BF 12–23 months (*n* 219)	
	AOR	95 % CI	AOR	95 % CI	AOR	95 % CI	AOR	95 % CI	AOR	95 % CI	AOR	95 % CI
Individual-level factors												
Maternal age (years)												
15–24	1·00		1·00		1·00		1·00		1·00		1·00	
25–34	0·40	0·11, 1·44	0·82	0·36, 1·88	0·62	0·26, 1·49	0·31	0·07, 1·51	2·52	0·51, 12·39	0·18	0·02, 2·21
35–49	0·15	0·02, 0·85	0·62	0·22, 1·72	0·57	0·21, 1·52	0·07	0·01, 0·74	4·33	0·18, 104·37	0·07	0·00, 1·14
Maternal education level												
Primary school or less			1·00		1·00							
Intermediate, high school or technical diploma			3·85	1·21, 12·19	0·61	0·14, 2·66						
University degree or higher			3·33	0·91, 12·18	1·30	0·23, 7·43						
Partner’s education level												
Primary school or less			1·00				1·00					
Intermediate, high school or technical diploma			1·30	0·61, 2·79			2·77	0·23, 33·96				
University degree or higher			4·67	1·57, 13·92			0·14	0·01, 3·15				
Maternal working status												
Working	1·00				1·00		1·00					
Not working	1·60	0·46, 5·54			0·63	0·27, 1·45	2·66	0·28, 25·29				
Partner’s working status												
Working									1·00			
Not working									4·37	0·10, 189·63		
Sex of the child												
Male	1·00		1·00		1·00		1·00		1·00		1·00	
Female	1·13	0·56, 2·28	1·50	0·77, 2·91	1·29	0·76, 2·17	0·55	0·11, 2·91	2·59	0·64, 10·48	0·91	0·22, 3·70
Birth order												
First born	1·00				1·00						1·00	
Second to fourth	3·88	1·53, 9·87			1·97	0·94, 4·13					5·31	0·66, 42·77
Fifth or more	13·77	2·42, 78·46			5·76	1·88, 17·70					10·12	0·87, 117·74
Mode of delivery												
Vaginal delivery	1·00		1·00		1·00		1·00		1·00		1·00	
Caesarean section delivery	0·65	0·30, 1·41	0·58	0·30, 1·12	0·99	0·57, 1·73	3·73	0·57, 24·50	0·84	0·26, 2·71	0·12	0·02, 0·66
Maternal BMI†												
Normal					1·00						1·00	
Underweight					7·15	0·66, 76·92						
Overweight					0·65	0·33, 1·28					0·98	0·17, 5·71
Obese					0·27	0·09, 0·79					5·80	0·77, 43·52
Maternal smoking												
Ever	1·00						1·00		1·00		1·00	
Never	0·33	0·13, 0·84					9·82	1·14, 84·37	0·21	0·05, 0·84	9·26	0·68, 125·58
Alcohol drinking												
Ever											1·00	
Never											8·49	0·50, 143·23
Tertiles of maternal knowledge score related to breast-feeding												
1 (lowest)	1·00		1·00		1·00		1·00		1·00		1·00	
2	1·82	0·46, 7·17	1·42	0·61, 3·29	1·75	0·69, 4·41	9·09	1·51, 54·81	0·39	0·07, 2·25	6·55	0·61, 70·48
3 (highest)	8·87	2·38, 33·09	1·35	0·61, 3·01	3·20	1·47, 6·94	5·41	0·58, 50·28	2·62	0·36, 19·11	7·09	2·14, 23·55
Household-level factors												
Household monthly income (Lebanese Pounds)												
<1 000 000			1·00		1·00						1·00	
1 000 000–2 000 000			1·16	0·54, 2·51	1·18	0·48, 2·87					0·68	0·07, 7·06
>2 000 000			1·16	0·41, 3·27	1·18	0·39, 3·53					3·45	0·40, 30·10
Doesn’t Know/refused to answer			2·10	0·97, 4·52	0·69	0·31, 1·53					4·97	0·98, 25·22
Presence of paid helper in the household												
No	1·00		1·00		1·00		1·00		1·00		1·00	
Yes	0·43	0·16, 1·20	2·05	0·67, 6·30	1·87	0·88, 3·99	1·05	0·12, 9·39	0·62	0·14, 2·67	0·02	0·00, 0·64
Partner providing positive support for breast-feeding												
No (including neutral)	1·00		1·00		1·00		1·00		1·00		1·00	
Yes	6·70	2·64, 17·01	2·53	0·86, 7·43	2·62	0·73, 9·43	0·16	0·02, 1·16	3·22	0·34, 30·04	0·13	0·02, 0·77
Community-level factors												
Governorate†												
Beirut											1·00	
Mount Lebanon												
North											1·10	0·13, 9·16
South and Nabatiyeh											3·59	0·22, 57·43
Bekaa											0·50	0·06, 3·90
Hospital providing support for breast-feeding your child after delivery												
Yes	1·00		1·00		1·00		1·00		1·00		1·00	
No/Neutral	0·44	0·15, 1·32	1·22	0·51, 2·94	0·89	0·35, 2·24	7·31	0·68, 78·72	0·47	0·07, 2·99	0·75	0·17, 3·45

* The independent variables which were entered in each of the multiple regression models were those that were associated with the outcome at a *P*-value lower than 0 2 and/or variables that are deemed important according to literature. The variables selected and entered in the multiple regression models for each of the IYCF practices are displayed in the table.

† For the outcome ‘Continued BF 12–23 months’, the variables ‘maternal BMI’ and ‘Governorates’ were regrouped given the small number of counts in cells. For the maternal BMI: normal and underweight were grouped into one category which became the reference. For Governorate, Beirut and Mount Lebanon were grouped into one category which became the reference.

As for the household-level variables, the partner’s support for BF was associated with higher odds of ever BF (AOR = 6·70, *P* < 0·001) while the presence of a paid helper was associated with lower odds of continued BF at 12–23 months (AOR = 0·02, *P* = 0·027) (Table 4). The block of household-level variables was significant (*P* < 0·05) for the following BF indicators: ever BF, EBF for the first 2 d and mixed milk feeding under 6 months (online supplementary material, Supplemental Table 4). None of the community-level variables (or their corresponding block) was significantly associated with the studied BF indicators.

The results of the models describing the factors associated with CF indicators are presented in Tables 5 and 6, while their corresponding models’ statistics are shown in online supplementary material, Supplemental Table 4. Regarding the individual-level factors, maternal age and maternal overweight or obesity were associated with lower odds of meeting both the MDD and MAD indicators, while maternal underweight was associated with lower odds of meeting MDD (AOR = 0·04, *P* = 0·048) (Table 5). As compared with children aged 6–11 months, those aged 12–23 months were more likely to meet the MDD, MMF and MAD indicators (AOR = 2·54, 4·84 and 2·09 with *P* = 0·005, 0·043 and 0·033, respectively). Partner’s support for BF, as a household-level variable, was associated with higher odds of meeting MMF and MAD (AOR = 5·68 with *P* = 0·012 and AOR = 2·81 with *P* = 0·037, respectively). Amongst the community-level variables, residing in the Bekaa was associated with around five times the odds of meeting MAD (*P* = 0·010) (Table 5). The block of individual-level factors was significant for all of the three indicators (MDD, MMF and MAD) (*P* < 0·05) while that of the household-level variables was significant for MMF (*P* = 0·022) and MAD (*P* = 0·004) only. The community block did not show significance for any of the three indicators (online supplementary material, Supplemental Table 4).

**Table 5:** Adjusted odds ratios (AOR) and their corresponding 95 % CI for the association of various characteristics with different complementary feeding indicators among children, Lebanon 2012–2013*

Variables	Minimum dietary diversity (MDD: 5 out of 8) (*n* 367)		Minimum meal frequency (MMF) (*n* 367)		Minimum acceptable diet (MAD) (*n* 367)	
	AOR	95 % CI	AOR	95 % CI	AOR	95 % CI
Individual-level factors						
Maternal age (years)						
15–24	1·00		1·00		1·00	
25–34	0·39	0·14, 1·07	1·29	0·44, 3·82	0·38	0·14, 1·04
35–49	0·29	0·11, 0·74	1·70	0·37, 7·91	0·34	0·13, 0·88
Maternal working status						
Working			1·00			
Not working			0·19	0·03, 1·08		
Partner’s working status						
Working	1·00				1·00	
Not working	0·31	0·05, 1·91			0·28	0·04, 1·83
Sex of the child						
Male	1·00		1·00		1·00	
Female	0·85	0·47, 1·55	1·62	0·41, 6·42	1·01	0·55, 1·86
Child’s age (months)						
6–11	1·00		1·00		1·00	
12–23	2·54	1·33, 4·86	4·84	1·05, 22·24	2·09	1·06, 4·11
Birth order						
First born	1·00				1·00	
Second to fourth	2·06	0·95, 4·48			1·78	0·82, 3·86
Fifth or more	4·68	0·96, 22·91			3·29	0·66, 16·52
Maternal BMI						
Normal	1·00				1·00	
Underweight	0·04	0·00, 0·97			0·06	0·00, 1·20
Overweight	0·24	0·11, 0·51			0·27	0·12, 0·58
Obese	0·35	0·17, 0·74			0·43	0·21, 0·88
Maternal smoking						
Ever			1·00			
Never			0·10	0·02, 0·49		
Tertiles of maternal knowledge score related to breast-feeding						
1 (lowest)	1·00		1·00		1·44	
2	1·46	0·63, 3·38	0·42	0·08, 2·22	1·44	0·60, 3·48
3 (highest)	1·13	0·53, 2·42	0·70	0·18, 2·76	1·37	0·64, 2·92
Household-level factors						
Household monthly income (Lebanese Pounds)						
<1 000 000	1·00				1·00	
1 000 000–2 000 000	2·38	0·91, 6·21			2·13	0·78, 5·84
>2 000 000	1·70	0·57, 5·09			2·43	0·83, 7·13
Doesn’t Know/refused to answer	4·13	1·48, 11·51			5·07	1·72, 14·93
Presence of paid helper in the household						
No	1·00		1·00		1·00	
Yes	0·85	0·37, 1·93	1·26	0·31, 5·08	0·42	0·17, 1·03
Partner providing positive support for breast-feeding						
No (including neutral)			1·00		1·00	
Yes	2·28	0·96, 5·43	5·68	1·49, 21·63	2·81	1·06, 7·44
Community-level factors						
Governorate						
Beirut	1·00				1·00	
Mount Lebanon	2·23	0·52, 9·63			1·79	0·54, 5·90
North	1·30	0·30, 5·70			1·61	0·47, 5·59
South and Nabatiyeh	2·73	0·59, 12·72			2·73	0·76, 9·85
Bekaa	3·66	0·86, 15·66			4·81	1·46, 15·84
Hospital providing support for breast-feeding your child after delivery						
Yes	1·00		1·00		1·00	
No/Neutral	0·85	0·38, 1·93	0·39	0·12, 1·26	0·58	0·23, 1·41

* The independent variables which were entered in each of the multiple regression models were those that were associated with the outcome at a *P*-value lower than 0 2 and/or variables that are deemed important according to literature.

The variables selected and entered in the multiple regression models for each of the IYCF practices are displayed in the table.

**Table 6:** Adjusted OR (95 % CI) for the association of various characteristics with different complementary feeding indicators among children, Lebanon 2012–2013*

Variables	Introduction of solid, semi-solid or soft foods 6–8 months (*n* 74)		Egg and/or flesh food consumption 6–23 months (*n* 367)		Sweet beverage consumption 6–23 months (*n* 367)		Unhealthy food consumption 6–23 months (*n* 367)		No vegetable or fruit consumption 6–23 months (*n* 367)	
	OR	95 % CI	OR	95 % CI	OR	95 % CI	OR	95 % CI	OR	95 % CI
Individual-level factors										
Maternal age (years)										
15–24	1·00		1·00		1·00		1·00		1·00	
25–34	3·17	0·15, 68·85	0·47	0·18, 1·19	0·16	0·04, 0·57	0·82	0·37, 1·83	1·74	0·51, 5·91
35–49	0·38	0·03, 4·60	0·67	0·24, 1·85	0·17	0·05, 0·51	0·26	0·09, 0·73	1·89	0·46, 7·81
Maternal education level										
Primary school or less					1·00					
Intermediate, high school or technical diploma					1·49	0·57, 3·92				
University degree or higher					0·92	0·27, 3·21				
Partner’s education level										
Primary school or less			1·00						1·00	
Intermediate, high school or technical diploma			1·15	0·57, 2·32					0·55	0·19, 1·54
University degree or higher			2·02	0·90, 4·51					0·40	0·08, 2·10
Maternal working status										
Working									1·00	
Not working									1·74	0·61, 4·94
Sex of the child										
Male	1·00		1·00		1·00		1·00		1·00	
Female	1·94	0·26, 14·62	1·52	0·77, 3·01	0·92	0·51, 1·65	1·52	0·83, 2·76	0·66	0·30, 1·43
Child’s age (months)										
6–11			1·00		1·00		1·00		1·00	
12–23			3·79	1·95, 7·38	3·64	1·48, 8·97	7·12	3·24, 15·64	0·28	0·13, 0·59
Birth order										
First born					1·00				1·00	
Second to fourth					4·45	2·22, 8·99			0·27	0·11, 0·69
Fifth or more					5·48	1·72, 17·52			1·26	0·32, 4·93
Mode of delivery										
Vaginal delivery					1·00					
Caesarean section delivery					0·66	0·40, 1·11				
Maternal BMI										
Normal			1·00							
Underweight			2·61	0·11, 60·21						
Overweight			0·48	0·21, 1·09						
Obese			0·42	0·19, 0·93						
Maternal smoking										
Ever					1·00					
Never					0·44	0·22, 0·91				
Alcohol drinking										
Current alcohol drinker									1·00	
Not alcohol drinker									2·08	0·45, 9·55
Household-level factors										
Household monthly income (Lebanese Pounds)										
<1 000 000			1·00		1·00				1·00	
1 000 000–2 000 000			1·83	0·72, 4·66	0·53	0·19, 1·42			0·38	0·18, 0·80
>2 000 000			1·89	0·52, 6·85	0·75	0·25, 2·30			0·43	0·12, 1·56
Doesn’t know/refused to answer			1·38	0·54, 3·52	0·86	0·35, 2·10			0·37	0·13, 1·07
Presence of paid helper in the household										
No	1·00		1·00		1·00		1·00		1·00	
Yes	0·20	0·02, 2·18	0·57	0·23, 1·43	0·65	0·32, 1·32	0·70	0·24, 2·10	0·52	0·13, 2·14
Community-level factors										
Governorate										
Beirut			1·00		1·00				1·00	
Mount Lebanon			2·85	1·18, 6·90	0·25	0·10, 0·62			1·16	0·13, 10·45
North			1·57	0·55, 4·43	0·47	0·20, 1·07			2·24	0·27, 18·69
South and Nabatiyeh			2·94	1·11, 7·83	0·26	0·08, 0·79			0·66	0·06, 6·77
Bekaa			2·81	1·13, 7·01	0·68	0·26, 1·77			0·39	0·03, 5·71

* The independent variables which were entered in each of the multiple regression models were those that were associated with the outcome at a *P*-value lower than 0·2 and/or variables that are deemed important according to literature.

The variables selected and entered in the multiple regression models for each of the IYCF practices are displayed in the table.

With regard to the remaining CF indicators and their association with individual-level factors (Table 6), the results showed that higher maternal age was associated with lower odds of consuming sweet beverages (AOR = 0·16 with *P* = 0·006 and AOR = 0·17 with *P* = 0·002 for maternal age 25–34 years and 35–49 years, respectively) and unhealthy food amongst children (AOR = 0·26 for maternal age 35–49 years, *P* = 0·011). Moreover, compared with 6–11-month-old children, those aged 12–23 months were more likely to consume sweet beverages (AOR = 3·64, *P* = 0·005), unhealthy food (AOR = 7·12, *P* < 0·001) or eggs and/or flesh food (AOR = 3·79, *P* < 0·001); older children (12–23 months) were also less likely to have no fruit or vegetable consumption (AOR = 0·28, *P* = 0·001). Moreover, children whose mothers were obese had lower odds of consuming egg and/or flesh food than those whose mothers had a normal BMI (AOR = 0·42, *P* = 0·032). The results also showed that higher birth order was associated with greater odds of consuming sweet beverages (AOR = 4·45 with *P* < 0·001 for second to fourth birth order and 5·48 with *P* = 0·005 for fifth or more birth order), while maternal non-smoking was associated with lower odds (AOR = 0·44, *P* = 0·028). Lastly, second to fourth born children had lower odds of having no vegetable or fruit consumption, compared with first-born children (AOR = 0·27, *P* = 0·007).

Amongst the household-level variables, middle income (1 000 000–2 000 000 L.L) was associated with lower odds of having no fruits or vegetables consumption, compared with lower income (AOR = 0·38, *P* = 0·011). As for community-level factors, the geographical area of residence was found to be associated with two indicators: compared with those living in Beirut, children living in other governorates had approximately three times the odds of consuming egg/flesh food and lower odds of sweet beverage consumption (AOR = 2·85 for Mount Lebanon with *P* = 0·021 and 0·26 for South and Nabatiyeh with *P* = 0·019) (Table 6). The block of individual-level variables was significant for all of the indicators shown in Table 6 (*P* < 0·001), except for the introduction of solid food. The household block was only significant for the indicator pertinent to unhealthy food consumption (*P* < 0·001), while the community block was significant for the indicators related to the consumption of sweet beverages and vegetables/fruits (*P* = 0·046 and 0·047, respectively) (online supplementary material, Supplemental Table 4).

For certain measures of associations examined in this study, the CI were wide, such is the case for the association between continued BF at 12–23 months and smoking status of the mother (AOR: 9·26, 95 % CI (0·68, 125·58)). The wide CI was due to the low count of observations within cells when the dependent and independent variables were cross-tabulated: among mothers who continued to BF, the number of current smokers was only ‘4’.

## Discussion

To our knowledge, this was the first study from the EMR to investigate BF and CF practices, using the most recent IYCF indicators. It showed that, while the majority of mothers have reported ever BF (87·6 %), the proportion of infants aged less than 6 months who were exclusively breastfed was low not exceeding 11 %. This discrepancy between the high prevalence of ever BF and the low prevalence of EBF amongst 0–5 months infants was previously described in Lebanon (89 % for ever BF *v*. 10 % for EBF)^(6)^, Kuwait (89·9 % *v*. 8 %)^(23)^, Qatar (97·9 % *v*. 18·9 %)^(24)^ and Canada (90·3 % *v*. 14·4 %)^(25)^, while the discrepancy was narrower in Turkey (96·7 % *v*. 41·6 %)^(25)^. The comparison between studies may be limited by methodological disparities in the assessment of EBF (e.g. retrospective recall *v*. current practices) and hence ought to be interpreted with caution. The observed prevalence of EBF in infants aged less than 6 months was in line with previous reports from the country^(6,8)^, and placed Lebanon amongst countries with the lowest rates worldwide^(24–26)^. This low prevalence in Lebanon may be a direct reflection of the introduction of other types of milk and/or soft/solid food early in life^(5)^. Our study findings showed that 45·2 % of 0–5 months infants were not breastfed at all (data not shown) and 42·1 % were receiving mixed milk feeding (formula in addition to breast milk), while 22·8 % of 4–5 months olds were already receiving solid, semi-solid or soft food (data not shown). These findings are of concern given the evidence linking EBF to decreased infant mortality, particularly in low- and middle-income countries^(27)^. Several studies have also reported a significantly lower risk of diarrhoea, respiratory infections, sepsis and other infections amongst exclusively breastfed infants in the first few months of life, compared with those receiving partial BF^(27)^. In addition, EBF was suggested to protect against childhood obesity^(28)^, a condition that is highly prevalent in Lebanon^(17)^ and other countries of the EMR^(4)^.

Early BF initiation was found to be suboptimal in our study. The WHO and UNICEF reported in 2017 that only about two in five infants (42 %) worldwide were put to the breast within the first hour of life^(29)^. The prevalence rate observed in Lebanon (28 %) was lower than this global estimate, while being also lower that the value of 35 % that was reported for the EMR^(29)^. In Lebanon, hospitals often do not comply with the WHO’s ten steps of the Baby-Friendly Hospital Initiative, and the separation of mother and infants is commonly practised, with formula milk being available and often used to feed infants^(30)^. Acknowledging that early BF initiation is crucial for establishing BF over the long term, and predicting future EBF^(31)^, the low prevalence of this practice may explain the observed low rates of EBF during the first 2 d after birth (28·6 %) as well the low prevalence of EBF amongst 0–5 months infants. Early initiation of BF has also been linked with improved newborn’s survival^(31)^, thus highlighting the need for policy and programmatic action aimed at promoting this practice in Lebanon.

The investigation of factors that are potentially associated with BF practices in Lebanon was performed at three levels in our study: individual, household and community. At the individual level, higher education amongst mothers was associated with greater odds of early BF initiation in the study sample. Victor *et al.* ^(13)^ had also shown that delayed initiation of BF was associated with lower maternal education, while Banu and Khanom^(32)^ showed that higher maternal education was associated with higher BF knowledge. Our findings may be explained by the theory of planned behaviour and reasoned action, whereby mothers with higher BF knowledge were more likely to adhere to adequate BF practices as a routine behaviour^(33)^. In this study, a greater BF knowledge amongst mothers was associated with higher odds of ever BF, EBF during the first 2 d after birth, EBF for up to 6 months and continued BF in children aged 12–23 months. These results were in line with those reported by other studies highlighting BF knowledge as a key modulator of BF behaviour and underlined the need for proper interventions aimed at raising maternal knowledge about the benefits of BF and combatting misconceptions^(33–36)^. Mothers whose child had a higher birth order were also found to be more likely to ever breastfeed and to exclusively breastfeed during the first 2 d after birth. A study in Tanzania showed that first-time mothers tend to have more difficulties in establishing BF and are more likely to be anxious and self-doubting compared with mothers who had previous experience with other children^(13)^. Our study findings also showed that non-smoking mothers had significantly higher odds of EBF for up to 6 months as well as lower odds of mixed milk feeding in infants aged less than 6 months. Previous studies have reported maternal smoking as an important adverse factor for the exclusivity and duration of BF^(37)^. These observations may be due to the effect of tobacco smoke on lactation leading to decreased quantity of milk production^(38)^ that may limit the capacity of women to exclusively breastfed their infant. Moreover, our results showed that maternal obesity was associated with lower odds of EBF for the first 2 d, a finding that is consistent with that reported by several other studies^(7,8,39)^, where maternal obesity was found to be associated with significantly lower rates of BF initiation, duration and/or exclusivity^(39)^. The factors that may impact early BF in obese women may include mechanical factors as well as delayed onset of lactogenesis II^(39)^.

In our study, the education level of the partner was also found to be an independent predictor of early BF initiation, an observation that highlighted the important role that fathers/partners may play in encouraging BF^(40)^. A study conducted in Norway^(41)^ showed that higher paternal education was associated with EBF at 4 months of age, and in the Netherlands, Lanting *et al.* ^(42)^ showed that women who had a higher-educated partner were more likely to initiate BF. It was argued that, by equipping fathers/partners with adequate knowledge about BF benefits, complications and cues, they will have the tools to deal with the often encountered feelings of being helpless or left-out, while also allowing them to better support the mother^(42)^. Interestingly, in our study, and amongst the investigated household-level factors, partner’s support for BF was found as an independent predictor of ever BF, increasing its odds by approximately seven folds. In a study conducted amongst partners of women who gave birth in the previous 2 years, Brown and Davis^(43)^ have highlighted the need for programmes that direct support and information towards fathers as well as mother–infant dyads, in recognition of the important role that fathers can play in BF enabling. Another household-level factor that was found to be linked with BF practices in our study was the presence of a live-in paid helper, which was associated with significantly lower odds of continued BF at 12–23 months. Based on available data in 2010, it was estimated that Lebanon hosted 117 941 paid sleep-in domestic workers who come from foreign countries and live in the employer’s house for the duration of their contract^(44)^. It is therefore possible that mothers have delegated the responsibility of child feeding to the helper, which essentially would consist of formula feeding, hence the observed inverse relationship with continued BF.

As for CF practices in Lebanon, the majority of 6–8-month-old infants were found to be already introduced to solid, semi-solid or soft food (96·2 %). This value was close to that reported from Tanzania (92·3 %)^(16)^ and Zambia (90 %)^(26)^, while being higher than estimates reported from other countries such as Kuwait (69 %)^(23)^, Nepal (69·7 %)^(14)^, Ethiopia (60·7 %)^(26)^ and Pakistan (39·2 %)^(15)^. Similarly, the majority of 6–23-month-old children participating in the study (92·8 %) were found to meet the MMF indicator, an estimate that was higher than that reported from several other countries such as Nepal (82 %)^(14)^, Ethiopia (54·7 %)^(26)^, UAE (47 %)^(45)^, Pakistan (38 %)^(46)^ and Tanzania (34·2 %)^(16)^. The observed high proportions of children meeting MMF and the timely introduction of solid, semi-solid or soft food suggested that Lebanese mothers comply with the WHO guidelines related to the recommended frequency of feeding. However, the study findings showed that dietary diversity and the quality of the diet were suboptimal with only close to a third of children meeting the MDD and MAD indicators. The prevalence of those meeting MDD in Lebanon (55·8 %) was higher than that reported by Shaker-Berbari *et al.* ^(7)^, based on the UNICEF survey conducted in 2016 (26 %), and where non-quantitative data on food consumption were collected. The prevalence in our study was relatively similar to that reported from Kuwait (41·6 %)^(23)^, Tanzania (38·2 %)^(16)^ and Zambia (37·4 %)^(26)^ but lower than that reported from the UAE (71·1 %)^(45)^. The MAD indicator, which is a composite score of both MDD and MMF, was also assessed in our study, with an overall prevalence of 34·4 %. This value was similar to that reported from the UAE (36·2 %)^(45)^, but higher than that described in Tanzania (15·9 %)^(16)^, Pakistan (12 %)^(46)^ and Zambia (25·1 %)^(26)^. Some of the factors that may explain inter-country differences in CF practices include socio-economic factors such as cultural habits and beliefs, household income and poverty level, maternal education and literacy as well as lack of awareness on adequate CF practices^(47)^. In addition, and based on the recent IYCF indicators, we have assessed the MAD indicator for breastfed and non-breastfed children separately, and this analysis documented a higher proportion of children meeting MAD in the breastfed category (49·4 % *v*. 29·7 %). The observed difference may be a reflection of the different definitions of MAD based on the child’s BF status, whereby for non-breast-fed infants, MAD was defined as receiving at least two milk feeds in addition to the MDD and MMF for their age^(11)^.

When examining the types of complementary foods, it became clear that the highest level of consumption was for grains, roots and tubers, which tend to have low nutrient density, especially that in Lebanon, the vast majority of grains are commonly consumed in the refined form^(16,48)^. At the same time, the food groups with the lowest consumption included pulses and vitamin A rich fruits and vegetables, which are typically rich in dietary fibre, phytochemicals and antioxidants and are known to confer numerous health protective properties^(49)^. In addition, approximately one fifth of children aged 6–23 months had no vegetable or fruit consumption during the previous day. This finding, when coupled with the fact that half of the children had consumed an ‘unhealthy food’ and more than a third had consumed a sweetened beverage on the day preceding the interview, suggested that dietary quality and diversity in this age group may be inadequate to provide sufficient amounts of nutrients, support optimal growth and prevent obesity^(50)^. These findings highlighted the need for effective interventions to educate mothers, caregivers, healthcare professionals and the communities as a whole on how to improve the quality of complementary foods in Lebanon.

The odds of meeting MDD, MMF and MAD were higher amongst older children, compared with those aged 6–11 months. These findings were in line with those reported from several other countries such as the Philippines, Pakistan and Nepal^(51–53)^. At the global level, less than 25 % of children aged 6–11 months of age were found to receive four or more food groups/d, whereas close to half of older children aged 18–23 months were reported to receive more than four food groups a day^(54)^. These observations may be a reflection of maternal perceptions towards IYCF, whereby mothers may think that children before the age of 1 year should not consume foods like pulses or eggs and flesh food^(53)^. Strategies and interventions targeting mothers with younger children and aimed at promoting dietary diversity and optimal feeding practices are therefore needed. Conversely, the odds of consuming sweetened beverages and unhealthy food groups were also found to be higher amongst older children compared with those aged 6–11 months, which was in agreement with previous studies reported in the literature^(55)^. Our results have also shown that older maternal age was associated with a lower likelihood of sweet beverage and unhealthy food consumption amongst children, a fact that may reflect a higher level of experience amongst older mothers^(7)^. Finally, our study showed that maternal obesity was associated with lower odds of meeting both the MDD and MAD indicators. This finding was similar to that reported by Mulaw *et al.* ^(56)^ in Northern Ethiopia, implying that mothers with a high BMI may be engaging, themselves, in unhealthy dietary practices, which also affect their feeding practices and hence their child’s diet^(56)^.

At the household level, the finding that higher income was associated with lower odds of having no fruit/vegetable consumption suggested a possible inverse socio-economic gradient in healthy eating, which was previously described in the literature^(7)^. As for the community-level factors, our study showed that residing in the geographical area of the Bekaa, which is the main agricultural area of Lebanon, was associated with higher odds of meeting MAD. This may be explained by the higher accessibility and affordability of locally grown agricultural produce, which in turn may have a direct impact on food consumption habits^(57)^. Similarly, compared with children living in Beirut, the capital and urban centre of Lebanon, those living in other geographical areas had lower odds of consuming sweetened beverages, a fact that was in line with the literature on urban living and its potential impact on beverage consumption^(58)^.

The main strengths of this study comprised the use of nationally representative survey data, the adoption of the newly updated definitions of IYCF indicators, the investigation of factors that may be associated with suboptimal IYCF practices and the use of the WHO protocol in data collection, which circumvents the limitations of the retrospective recall approach^(12)^. The findings of this study ought to be considered in light of the following limitations. The investigation of IYCF practices were based on a 24-h recall rather than a longer recall period. This short recall may have resulted in missing some infants who were fed other liquids or foods prior to the 24 h before the survey. Therefore, the estimates obtained in this study may represent a best-case scenario, and actual population parameters for BF could be even lower, thus further highlighting the need for immediate action in improving IYCF in Lebanon. In addition, the number of community-level factors that were investigated for their association with IYCF was small, given that the initial survey collected few of these variables. Future studies addressing community-level determinants of IYCF are encouraged to include additional factors such as community-level access to antenatal and postnatal care, community poverty level, community’s media exposure and community-based BF promotion, support and advocacy^(59)^. Finally, it may be argued that the time that has elapsed between data collection (2012–2013) and the data analysis undertaken in this study (early 2021) may raise questions on whether the study findings are reflective of the current situation in the country. However, during this period, there has been no implementation or development of health or nutrition policies/programmes aimed at improving IYCF in Lebanon. Hence, it is possible that no major improvements in IYCF practices have occurred during the elapsed period. Alternatively, it may be argued that infant feeding indicators may have worsened during the past 10 years, as has been observed in other countries^(47,60)^. For example, increased supply and advertising of ultra-processed foods, no or poor implementation of the Baby Friendly Hospital Initiative and the intensification of marketing strategies that discourage BF^(61,62)^ may have contributed towards a further deterioration of IYCF practices in Lebanon.

In conclusion, this study has characterised, for the first time, IYCF practices amongst Lebanese children and identified factors that may impact or modulate these practices. The results showed low rates of early initiation of BF and of EBF, while also highlighting suboptimal CF practices with low dietary diversity and frequent consumption of sweeteners beverages and other foods high in sugar, salt and/or unhealthy fats. Findings from this study will help guide the development of culture-specific interventions and programmes aimed at improving BF and CF practices in Lebanon. In particular, these programmes should target first-time mothers, the least educated mothers, smokers and those with obesity, in addition to focusing on fathers/partners given the support that they can offer to BF mothers. The improvement of IYCF practices will require national commitment to foster progress towards the 2025 global nutrition target of increasing BF in Lebanon.

## Supplementary material

For supplementary material accompanying this paper visit https://doi.org/ 10.1017/S1368980022000842. click here to view supplementary material
