# Supplementary material for: Infant and young child feeding practices in Lebanon: a cross-sectional national study
Source: Public Health Nutr. 2022 Apr 4;26(1):143–59. doi: 10.1017/S1368980022000842 (PMC11077449; doi:10.1017/S1368980022000842)
Supplement: Supplementary file 1 [file S1368980022000842sup001.docx]

**Supplementary Table 1. Definition and calculation of the various IYCF indicators as per the WHO/UNICEF 2021 guidance document.**

| **Indicator** | **Definition** |
| --- | --- |
| Ever breastfed | Proportion of children born in the last 24 months who were ever breastfed. |
| Early initiation of breastfeeding | Proportion of children born in the last 24 months who were put to the breast within one hour of birth. |
| Exclusively breastfed for the first two days after birth | Proportion of children born in the last 24 months who were fed exclusively with breast milk for the first two days after birth. |
| Exclusive breastfeeding under 6 months | Proportion of infants 0–5 months of age who were fed exclusively with breast milk during the previous day. |
| Mixed feeding under 6 months | Proportion of infants 0–5 months of age who were fed formula and/or animal milk in addition to breast milk during the previous day. |
| Continued breastfeeding 12-23 months | Proportion of children 12–23 months of age who were fed breast milk during the previous day. |
| Introduction of solid, semi-solid or soft foods 6-8 months | Proportion of infants 6–8 months of age who consumed solid, semi-solid or soft foods during the previous day. |
| Minimum dietary diversity 6-23 months | Proportion of children 6–23 months of age who consumed foods and beverages from at least 5 out of 8 defined food groups during the previous day.  The eight food groups used for computing this indicator include: Breast milk; Grains, roots, and tubers; Pulses (beans, peas, lentils), nuts and seeds; Dairy products (milk, infant formula, yogurt, cheese); Flesh foods; Eggs; Vitamin A rich fruits and vegetables, and other fruits and vegetables.  Calculation of minimum dietary diversity was based on a quantitative 24-hour recall. All recipes were disaggregated and data pertinent to the various ingredients was used for the calculation of minimum dietary diversity. For this indicator, the consumption of any amount of food or beverage from a specific food group is sufficient to “count”, i.e. there is no minimum quantity. |
| Minimum meal frequency 6-23 months | Proportion of children 6–23 months of age who consumed solid, semi-solid or soft foods (but also including milk feeds for non-breastfed children) the minimum number of times or more during the previous day.  The minimum number of times was defined as: two feedings of solid, semi-solid or soft foods for breastfed infants aged 6–8 months, three feedings of solid, semi-solid or soft foods for breastfed children aged 9–23 months, and four feedings of solid, semi-solid or soft foods or milk feeds for non-breastfed children aged 6–23 months whereby at least one of the four feeds must be a solid, semi-solid or soft feed. |
| Minimum milk feeding frequency for non-breastfed children 6-23 months | Percentage of non-breastfed children 6-23 months of age who consumed at least two milk feeds during the previous day. |
| Minimum acceptable diet 6-23 months | Proportion of children 6–23 months of age who consumed a minimum acceptable diet during the previous day.  This composite indicator is calculated from the following two fractions:   - breastfed children 6–23 months of age receiving at least the minimum dietary diversity and the minimum meal frequency for their age during the previous day, - and non-breastfed children 6–23 months of age receiving at least the minimum dietary diversity and the minimum meal frequency for their age during the previous day as well as at least two milk feeds. |
| Egg and/or flesh food consumption 6-23 months | Proportion of children 6-23 months of age who consumed egg and/or flesh food during the previous day. |
| Sweet beverage consumption 6-23 months | Proportion of children 6-23 months of age who consumed a sweet beverage during the previous day. |
| Unhealthy food consumption 6-23 months | Proportion of children 6-23 months of age who consumed selected sentinel unhealthy foods during the previous day.  “Sentinel unhealthy foods” are foods or categories of foods (e.g. “sweets” or “candies”) that are likely to be consumed by infants and young children and are high in sugar, salt and/or unhealthy fats.  • Selected sentinel unhealthy foods are:  – Candies, chocolate and other sugar confections, including those made with real fruit or vegetables like candied fruit or fruit roll-ups.  – Frozen treats like ice cream, gelato, sorbet, popsicles or similar confections.  – Cakes, pastries, sweet biscuits and other baked or fried confections which have at least a partial base of a refined grain, including those made with real fruit or vegetables or nuts, like apple cake or cherry pie.  – Chips, crisps, cheese puffs, French fries, fried dough, instant noodles and similar items which contain mainly fat and carbohydrate and have at least a partial base of a refined grain or tuber. These foods are also often high in sodium. |
| No vegetable or fruit consumption 6-23 months | Proportion of children 6-23 months of age who did not consume any vegetables or fruits during the previous day. |

Supplementary Table 2: Breastfeeding indicators across individual-, household- and community-level factors

|  | Ever Breastfed (n=469) | | | Early Initiation of Breastfeeding (n=469) | | | Exclusively breastfed for the first two days (n=469) | | | Exclusive breastfeeding under 6 months (n=102) (among 0-5.9 months) | | | Mixed milk feeding under 6 months (n=102) (among 0-5 months) | | | Continued BF 12-23 months (n=219) | | |
| --- | --- | --- | --- | --- | --- | --- | --- | --- | --- | --- | --- | --- | --- | --- | --- | --- | --- | --- |
|  | **OR** | **95% CI** | **p-value** | **OR** | **95% CI** | **p-value** | **OR** | **95% CI** | **p-value** | **OR** | **95% CI** | **p-value** | **OR** | **95% CI** | **p-value** | **OR** | **95% CI** | **p-value** |
| **Individual-level factors** | | | | | | | | | | | | | | | | | | |
| Maternal age (years) |  |  |  |  |  |  |  |  |  |  |  |  |  |  |  |  |  |  |
| 15-24 | *1.00* |  | 0.431 | *1.00* |  | 0.731 | *1.00* |  | 0.661 | *1.00* |  | 0.122 | *1.00* |  | 0.312 | *1.00* |  | 0.762 |
| 25-34 | 0.92 | 0.34, 2.46 |  | 0.99 | 0.41, 2.36 |  | 1.36 | 0.62, 2.96 |  | 0.32 | 0.06, 1.68 |  | 1.76 | 0.36, 8.55 |  | 0.65 | 0.18, 2.39 |  |
| 35-49 | 0.52 | 0.16, 1.71 |  | 0.73 | 0.30, 1.75 |  | 1.39 | 0.63, 3.08 |  | 0.10 | 0.01, 0.93 |  | 4.53 | 0.65, 31.75 |  | 0.88 | 0.13, 6.23 |  |
| Maternal education level |  |  |  |  |  |  |  |  |  |  |  |  |  |  |  |  |  |  |
| Primary school or less | *1.00* |  | 0.379 | *1.00* |  | 0.002 | *1.00* |  | 0.026 | *1.00* |  | 0.656 | *1.00* |  | 0.853 | *1.00* |  | 0.807 |
| Intermediate, high school, or Technical Diploma | 1.87 | 0.71 ,4.91 |  | 5.65 | 1.90, 16.80 |  | 0.84 | 0.24, 2.91 |  | 1.55 | 0.14, 17.13 |  | 1.58 | 0.15, 17.06 |  | 1.10 | 0.21, 5.85 |  |
| University degree or higher | 1.32 | 0.37, 4.72 |  | 6.15 | 2.30, 16.42 |  | 1.92 | 0.51, 7.20 |  | 2.58 | 0.27, 24.58 |  | 2.00 | 0.17, 23.22 |  | 0.62 | 0.08, 4.75 |  |
| Partner’s education level |  |  |  |  |  |  |  |  |  |  |  |  |  |  |  |  |  |  |
| Primary school or less | *1.00* |  | 0.824 | *1.00* |  | 0.025 | *1.00* |  | 0.881 | *1.00* |  | 0.083 | *1.00* |  | 0.486 | *1.00* |  | 0.832 |
| Intermediate, high school, or Technical Diploma | 0.91 | 0.36, 2.29 |  | 1.95 | 0.71, 5.33 |  | 0.91 | 0.39, 2.09 |  | 2.99 | 0.50, 17.90 |  | 0.81 | 0.12, 5.59 |  | 0.80 | 0.27, 2.30 |  |
| University degree or higher | 1.29 | 0.32, 5.23 |  | 7.24 | 1.75, 30.02 |  | 1.18 | 0.35, 3.98 |  | 0.41 | 0.03, 5.21 |  | 0.28 | 0.02, 3.43 |  | 1.19 | 0.20, 7.00 |  |
| Maternal working status |  |  |  |  |  |  |  |  |  |  |  |  |  |  |  |  |  |  |
| Working | *1.00* |  | 0.152 | *1.00* |  | 0.313 | *1.00* |  | 0.030 | *1.00* |  | 0.181 | *1.00* |  | 0.780 | *1.00* |  | 0.971 |
| Not working | 2.12 | 0.76, 5.96 |  | 0.65 | 0.28, 1.51 |  | 0.43 | 0.20, 0.92 |  | 3.81 | 0.53, 27.5 |  | 1.25 | 0.26, 6.11 |  | 1.03 | 0.22, 4.78 |  |
| Partner’s working status |  |  |  |  |  |  |  |  |  |  |  |  |  |  |  |  |  |  |
| Working | *1.00* |  | 0.334 | *1.00* |  | 0.556 | *1.00* |  | 0.374 | *1.00* |  | 0.946 | *1.00* |  | 0.035 | *1.00* |  | 0.369 |
| Not working | 2.84 | 0.34, 23.95 |  | 1.65 | 0.31, 8.92 |  | 0.59 | 0.18, 1.92 |  | 1.08 | 0.11, 10.79 |  | 11.09 | 1.19, 102.99 |  | 0.39 | 0.05, 3.14 |  |
| Sex of the child |  |  |  |  |  |  |  |  |  |  |  |  |  |  |  |  |  |  |
| Male | *1.00* |  | 0.448 | *1.00* |  | 0.970 | *1.00* |  | 0.307 | *1.00* |  | 0.675 | *1.00* |  | 0.175 | *1.00* |  | 0.175 |
| Female | 1.32 | 0.64, 2.71 |  | 1.02 | 0.43, 2.43 |  | 1.32 | 0.77, 2.27 |  | 0.74 | 0.17, 3.12 |  | 2.57 | 0.65, 10.17 |  | 1.98 | 0.73, 5.32 |  |
| Child’s age (months) |  |  |  |  |  |  |  |  |  |  |  |  |  |  |  |  |  |  |
| 0–5 | *1.00* |  | 0.026 | *1.00* |  | 0.140 | *1.00* |  | 0.065 | *NA* | *NA* | - | *NA* | *NA* | - | *NA* | *NA* | - |
| 6–11 | 3.27 | 0.71, 14.93 |  | 0.30 | 0.09, 1.00 |  | 2.74 | 1.09, 6.87 |  | *NA* | *NA* |  | *NA* | *NA* |  | *NA* | *NA* |  |
| 12–23 | 0.73 | 0.18, 2.89 |  | 0.37 | 0.12, 1.09 |  | 1.49 | 0.58, 3.88 |  | *NA* | *NA* |  | *NA* | *NA* |  | *NA* | *NA* |  |
| Birth order |  |  |  |  |  |  |  |  |  |  |  |  |  |  |  |  |  |  |
| First-born | *1.00* |  | 0.010 | *1.00* |  | 0.801 | *1.00* |  | 0.124 | *1.00* |  | 0.544 | *1.00* |  | 0.934 | *1.00* |  | 0.007 |
| Second to fourth | 1.94 | 1.08, 3.47 |  | 1.03 | 0.45, 2.37 |  | 1.36 | 0.63, 2.95 |  | 0.61 | 0.12, 3.03 |  | 0.81 | 0.21, 3.15 |  | 3.87 | 0.71, 21.02 |  |
| Fifth or more | 6.61 | 1.63, 26.82 |  | 1.38 | 0.53, 3.58 |  | 2.95 | 1.05, 8.30 |  | - |  |  | 1.22 | 0.11, 13.09 |  | 23.50 | 3.45, 160.03 |  |
| Mode of delivery |  |  |  |  |  |  |  |  |  |  |  |  |  |  |  |  |  |  |
| Vaginal delivery | *1.00* |  | 0.218 | *1.00* |  | 0.056 | *1.00* |  | 0.953 | *1.00* |  | 0.244 | *1.00* |  | 0.687 | *1.00* |  | 0.029 |
| Cesarean section delivery | 0.61 | 0.28, 1.35 |  | 0.45 | 0.20, 1.02 |  | 0.98 | 0.57, 1.70 |  | 2.41 | 0.54, 10.80 |  | 1.32 | 0.34, 5.17 |  | 0.27 | 0.08, 0.87 |  |
| Maternal Body Mass Index |  |  |  |  |  |  |  |  |  |  |  |  |  |  |  |  |  |  |
| Normal | *1.00* |  | 0.837 | *1.00* |  | 0.411 | *1.00* |  | 0.083 | *1.00* |  | 0.912 | *1.00* |  | 0.895 | *1.00* |  | 0.118 |
| Underweight | 0.58 | 0.05, 6.65 |  | 2.63 | 0.25, 27.65 |  | 2.25 | 0.24, 20.94 |  | - |  |  | - |  |  | - |  |  |
| Overweight | 1.33 | 0.62, 2.84 |  | 1.46 | 0.58, 3.66 |  | 0.63 | 0.32, 1.23 |  | 0.83 | 0.11, 6.05 |  | 0.94 | 0.18, 4.98 |  | 0.82 | 0.23, 2.98 |  |
| Obese | 1.19 | 0.41, 3.40 |  | 0.71 | 0.33, 1.53 |  | 0.34 | 0.14, 0.84 |  | 1.19 | 0.13, 10.84 |  | 1.38 | 0.30, 6.42 |  | 3.20 | 0.85, 12.02 |  |
| Maternal smoking |  |  |  |  |  |  |  |  |  |  |  |  |  |  |  |  |  |  |
| Current/Ex smoker | *1.00* |  | 0.117 | *1.00* |  | 0.654 | *1.00* |  | 0.464 | *1.00* |  | 0.111 | *1.00* |  | 0.016 | *1.00* |  | 0.012 |
| Non-smoker | 0.52 | 0.23, 1.18 |  | 1.17 | 0.59, 2.31 |  | 0.81 | 0.46, 1.42 |  | 3.17 | 0.76, 13.18 |  | 0.21 | 0.06, 0.74 |  | 6.53 | 1.53, 27.80 |  |
| Alcohol drinking |  |  |  |  |  |  |  |  |  |  |  |  |  |  |  |  |  |  |
| Current alcohol drinker | *1.00* |  | 0.221 | *1.00* |  | 0.984 | *1.00* |  | 0.827 | *1.00* |  | 0.310 | *1.00* |  | 0.561 | *1.00* |  | 0.008 |
| Not alcohol drinker | 1.74 | 0.71, 4.22 |  | 1.01 | 0.35, 2.94 |  | 1.14 | 0.35, 3.69 |  | 3.35 | 0.32, 35.44 |  | 2.02 | 0.18, 22.40 |  | 19.12 | 2.20, 165.96 |  |
| Tertiles of maternal knowledge score related to breastfeeding |  |  |  |  |  |  |  |  |  |  |  |  |  |  |  |  |  |  |
| 1 (lowest) | *1.00* |  | 0.152 | *1.00* |  | 0.317 | *1.00* |  | 0.025 | *1.00* |  | 0.971 | *1.00* |  | 0.179 | *1.00* |  | 0.183 |
| 2 | 1.71 | 0.43, 6.80 |  | 2.31 | 0.59, 9.04 |  | 1.25 | 0.46, 3.45 |  | 1.12 | 0.16, 8.07 |  | 0.37 | 0.05, 2.67 |  | 3.29 | 0.58, 18.56 |  |
| 3 (highest) | 3.26 | 0.96, 11.06 |  | 1.54 | 0.75, 3.15 |  | 3.03 | 1.34, 6.86 |  | 1.27 | 0.18, 9.13 |  | 2.65 | 0.60, 11.61 |  | 3.20 | 0.91, 11.22 |  |
| **Household-level factors** | | | | | | | | | | | | | | | | | | |
| Household monthly income (Lebanese Pounds) |  |  |  |  |  |  |  |  |  |  |  |  |  |  |  |  |  |  |
| <1,000,000 | *1.00* |  | 0.879 | *1.00* |  | 0.175 | *1.00* |  | 0.062 | *1.00* |  | 0.399 | *1.00* |  | 0.814 | *1.00* |  | 0.041 |
| 1,000,000-2,000,000 | 0.95 | 0.36, 2.53 |  | 1.18 | 0.53, 2.65 |  | 1.54 | 0.63, 3.78 |  | 1.37 | 0.25, 7.42 |  | 1.76 | 0.40, 7.69 |  | 0.21 | 0.03, 1.29 |  |
| >2,000,000 | 1.05 | 0.35, 3.16 |  | 2.06 | 0.84, 5.06 |  | 2.88 | 1.18, 7.03 |  | 0.49 | 0.06, 3.94 |  | 2.22 | 0.41, 11.95 |  | 1.19 | 0.15, 9.11 |  |
| Doesn’t Know/Refused to answer | 1.53 | 0.49, 4.78 |  | 2.78 | 0.88, 8.82 |  | 0.77 | 0.35, 1.66 |  | - |  |  | 1.51 | 0.13, 17.16 |  | 3.03 | 0.92, 9.93 |  |
| Presence of paid helper in the household |  |  |  |  |  |  |  |  |  |  |  |  |  |  |  |  |  |  |
| No | *1.00* |  | 0.069 | *1.00* |  | 0.499 | *1.00* |  | 0.062 | *1.00* |  | *0.857* | *1.00* |  | 0.906 | *1.00* |  | 0.004 |
| Yes | 0.46 | 0.20, 1.06 |  | 1.48 | 0.47, 4.66 |  | 1.93 | 0.97, 3.85 |  | 1.16 | 0.23, 5.97 |  | 1.10 | 0.22, 5.38 |  | 0.08 | 0.01, 0.43 |  |
| Partner providing positive support for breastfeeding |  |  |  |  |  |  |  |  |  |  |  |  |  |  |  |  |  |  |
| No | *1.00* |  | <0.001 | *1.00* |  | 0.211 | *1.00* |  | 0.092 | *1.00* |  | *0.962* | *1.00* |  | 0.880 | *1.00* |  | 0.285 |
| Yes | 5.40 | 2.18, 13.33 |  | 2.19 | 0.64, 7.55 |  | 2.38 | 0.87, 6.56 |  | 1.05 | 0.15, 7.18 |  | 0.84 | 0.09, 8.02 |  | 0.52 | 0.15, 1.75 |  |
| **Community-level factors** | | | | | | | | | | | | | | | | | | |
| Governorate |  |  |  |  |  |  |  |  |  |  |  |  |  |  |  |  |  |  |
| Beirut | *1.00* |  | 0.766 | *1.00* |  | 0.666 | *1.00* |  | 0.257 | *1.00* |  | 0.440 | *1.00* |  | 0.991 | *1.00* |  | 0.169 |
| Mount Lebanon | 0.49 | 0.14, 1.76 |  | 0.78 | 0.27, 2.26 |  | 0.80 | 0.35, 1.82 |  | 0.65 | 0.16, 2.66 |  | 1.28 | 0.18, 8.88 |  | 0.26 | 0.05, 1.27 |  |
| North | 0.74 | 0.20, 2.66 |  | 0.85 | 0.25, 2.88 |  | 0.49 | 0.20, 1.21 |  | 0.18 | 0.03, 1.27 |  | 1.17 | 0.15, 9.13 |  | 1.36 | 0.33, 5.55 |  |
| South and Nabatiyeh | 0.51 | 0.15, 1.78 |  | 0.59 | 0.20, 1.76 |  | 0.49 | 0.19, 1.24 |  | 0.37 | 0.08, 1.77 |  | 1.20 | 0.17, 8.42 |  | 1.65 | 0.34, 8.06 |  |
| Bekaa | 0.64 | 0.17, 2.44 |  | 1.15 | 0.39, 3.37 |  | 1.05 | 0.37, 2.98 |  | 0.33 | 0.02, 4.91 |  | 1.99 | 0.15, 26.23 |  | - |  |  |
| Hospital providing support for breastfeeding your child after delivery |  |  |  |  |  |  |  |  |  |  |  |  |  |  |  |  |  |  |
| Yes | *1.00* |  | 0.039 | *1.00* |  | 0.837 | *1.00* |  | 0.656 | *1.00* |  | 0.175 | *1.00* |  | 0.516 | *1.00* |  | 0.480 |
| No/Neutral | 0.37 | 0.15, 0.95 |  | 0.90 | 0.33, 2.45 |  | 0.82 | 0.35, 1.95 |  | 4.39 | 0.51, 37.83 |  | 0.52 | 0.07, 3.83 |  | 0.63 | 0.17, 2.31 |  |

OR, Odds Ratio; CI, Confidence Interval.

Supplementary Table 3a: Complementary feeding indicators across individual-, household- and community-level factors

|  | Introduction of solid, semi-solid or soft foods 6-8 months (n=74) | | | Minimum Dietary Diversity (MDD: 5 out of 8) (n=367) | | | Minimum Meal Frequency (MMF) (n=367) | | | Minimum Acceptable Diet (MAD) (n=367) | | |
| --- | --- | --- | --- | --- | --- | --- | --- | --- | --- | --- | --- | --- |
|  | **OR** | **95% CI** | **p-value** | **OR** | **95% CI** | **p-value** | **OR** | **95% CI** | **p-value** | **OR** | **95% CI** | **p-value** |
| **Individual-level factors** | | | | | | | | | | | | |
| Maternal age (years) |  |  |  |  |  |  |  |  |  |  |  |  |
| 15-24 | *1.00* |  | 0.321 | *1.00* |  | 0.818 | *1.00* |  | 0.469 | *1.00* |  | 0.861 |
| 25-34 | 1.88 | 0.09, 39.66 |  | 0.84 | 0.33, 2.19 |  | 1.55 | 0.25, 9.61 |  | 0.77 | 0.30, 2.00 |  |
| 35-49 | 0.29 | 0.02, 4.38 |  | 0.75 | 0.30, 1.92 |  | 2.97 | 0.44, 20.01 |  | 0.79 | 0.30, 2.04 |  |
| Maternal education level |  |  |  |  |  |  |  |  |  |  |  |  |
| Primary school or less | *1.00* |  | 0.485 | *1.00* |  | 0.944 | *1.00* |  | 0.505 | *1.00* |  | 0.232 |
| Intermediate, high school, or Technical Diploma | 4.10 | 0.37, 45.78 |  | 1.14 | 0.45, 2.85 |  | 2.06 | 0.54, 7.89 |  | 2.02 | 0.89, 4.57 |  |
| University degree or higher | 2.78 | 0.21, 36.57 |  | 1.02 | 0.36, 2.91 |  | 2.66 | 0.36, 19.77 |  | 1.60 | 0.56, 4.52 |  |
| Partner’s education level |  |  |  |  |  |  |  |  |  |  |  |  |
| Primary school or less | *1.00* |  | 0.810 | *1.00* |  | 0.277 | *1.00* |  | 0.207 | *1.00* |  | 0.333 |
| Intermediate, high school, or Technical Diploma | 0.74 | 0.06, 9.16 |  | 1.31 | 0.55, 3.14 |  | 3.57 | 0.85, 15.06 |  | 1.72 | 0.72, 4.15 |  |
| University degree or higher | - | - |  | 2.15 | 0.82, 5.62 |  | 1.95 | 0.28, 13.42 |  | 2.01 | 0.74, 5.49 |  |
| Maternal working status |  |  |  |  |  |  |  |  |  |  |  |  |
| Working | *1.00* |  | - | *1.00* |  | 0.758 | *1.00* |  | 0.066 | *1.00* |  | 0.709 |
| Not working | - | - |  | 1.14 | 0.50, 2.57 |  | 0.23 | 0.05, 1.10 |  | 1.17 | 0.51, 2.71 |  |
| Partner’s working status |  |  |  |  |  |  |  |  |  |  |  |  |
| Working | *1.00* |  | - | *1.00* |  | 0.097 | *1.00* |  | - | *1.00* |  | 0.080 |
| Not working | - | - |  | 0.33 | 0.09, 1.23 |  | - | - |  | 0.25 | 0.05, 1.18 |  |
| Sex of the child |  |  |  |  |  |  |  |  |  |  |  |  |
| Male | *1.00* |  | 0.597 | *1.00* |  | 0.594 | *1.00* |  | 0.452 | *1.00* |  | 0.244 |
| Female | 1.79 | 0.20, 16.22 |  | 1.22 | 0.58, 2.55 |  | 1.57 | 0.48, 5.19 |  | 1.55 | 0.74, 3.24 |  |
| Child’s age (months) |  |  |  |  |  |  |  |  |  |  |  |  |
| 0–5 | *NA* | *NA* | - | *NA* | *NA* |  | *NA* | *NA* |  | *NA* | *NA* |  |
| 6–11 | *NA* | *NA* |  | *1.00* |  | 0.005 | *1.00* |  | 0.241 | *1.00* |  | 0.024 |
| 12–23 | *NA* | *NA* |  | 2.57 | 1.33, 4.93 |  | 2.26 | 0.57, 8.93 |  | 2.15 | 1.11, 4.17 |  |
| Birth order |  |  |  |  |  |  |  |  |  |  |  |  |
| First-born | *1.00* |  | - | *1.00* |  | 0.132 | *1.00* |  | 0.449 | *1.00* |  | 0.135 |
| Second to fourth | - | - |  | 1.61 | 0.83, 3.09 |  | 2.44 | 0.60, 9.90 |  | 1.46 | 0.75, 2.84 |  |
| Fifth or more | - | - |  | 2.95 | 0.97, 8.99 |  | 1.28 | 0.23, 7.13 |  | 3.11 | 1.02, 9.49 |  |
| Mode of delivery |  |  |  |  |  |  |  |  |  |  |  |  |
| Vaginal delivery | *1.00* |  | 0.778 | *1.00* |  | 0.660 | *1.00* |  | 0.365 | *1.00* |  | 0.893 |
| Cesarean section delivery | 1.36 | 0.16, 11.76 |  | 0.87 | 0.45, 1.65 |  | 1.83 | 0.49, 6.88 |  | 0.96 | 0.50, 1.82 |  |
| Maternal Body Mass Index |  |  |  |  |  |  |  |  |  |  |  |  |
| Normal | *1.00* |  | 0.954 | *1.00* |  | 0.001 | *1.00* |  | 0.940 | *1.00* |  | 0.006 |
| Underweight | - | - |  | 0.10 | 0.01, 0.87 |  | - | - |  | 0.12 | 0.01, 1.09 |  |
| Overweight | 0.97 | 0.06, 15.44 |  | 0.23 | 0.10, 0.51 |  | 1.01 | 0.21, 4.80 |  | 0.26 | 0.12, 0.58 |  |
| Obese | 0.71 | 0.05, 10.65 |  | 0.43 | 0.19, 0.97 |  | 1.36 | 0.21, 8.93 |  | 0.52 | 0.23, 1.18 |  |
| Maternal smoking |  |  |  |  |  |  |  |  |  |  |  |  |
| Current/Ex smoker | *1.00* |  | 0.737 | *1.00* |  | 0.726 | *1.00* |  | 0.008 | *1.00* |  | 0.647 |
| Non-smoker | 0.66 | 0.06, 7.65 |  | 0.88 | 0.42, 1.83 |  | 0.15 | 0.04, 0.59 |  | 0.84 | 0.40, 1.78 |  |
| Alcohol drinking |  |  |  |  |  |  |  |  |  |  |  |  |
| Current alcohol drinker | *1.00* |  | 0.565 | *1.00* |  | 0.221 | *1.00* |  | 0.239 | *1.00* |  | 0.214 |
| Not alcohol drinker | 2.14 | 0.15, 29.98 |  | 1.61 | 0.75, 3.44 |  | 0.45 | 0.12, 1.71 |  | 1.61 | 0.76, 3.41 |  |
| Tertiles of maternal knowledge score related to breastfeeding |  |  |  |  |  |  |  |  |  |  |  |  |
| 1 (lowest) | *1.00* |  | 0.592 | *1.00* |  | 0.757 | *1.00* |  | 0.565 | *1.00* |  | 0.645 |
| 2 | 0.57 | 0.07, 4.64 |  | 1.32 | 0.61, 2.89 |  | 0.42 | 0.05, 3.39 |  | 1.34 | 0.60, 2.98 |  |
| 3 (highest) | - | - |  | 1.23 | 0.48, 3.15 |  | 0.53 | 0.13, 2.10 |  | 1.46 | 0.57, 3.78 |  |
| **Household-level factors** | | | | | | | | | | | | |
| Household monthly income (Lebanese Pounds) |  |  |  |  |  |  |  |  |  |  |  |  |
| <1,000,000 | *1.00* |  | 0.821 | *1.00* |  | 0.011 | *1.00* |  | 0.208 | *1.00* |  | 0.008 |
| 1,000,000-2,000,000 | 1.78 | 0.12, 26.38 |  | 3.07 | 1.17, 8.09 |  | 4.41 | 0.81, 24.08 |  | 2.45 | 0.88, 6.81 |  |
| >2,000,000 | - | - |  | 1.86 | 0.73, 4.77 |  | - | - |  | 1.95 | 0.73, 5.19 |  |
| Doesn’t Know/Refused to answer | 0.65 | 0.05, 9.27 |  | 4.81 | 1.95, 11.87 |  | 2.40 | 0.46, 12.57 |  | 5.07 | 2.00, 12.83 |  |
| Presence of paid helper in the household |  |  |  |  |  |  |  |  |  |  |  |  |
| No | *1.00* |  | 0.198 | *1.00* |  | 0.666 | *1.00* |  | 0.635 | *1.00* |  | 0.097 |
| Yes | 0.22 | 0.02, 0.26 |  | 0.80 | 0.29, 2.20 |  | 0.74 | 0.22, 2.56 |  | 0.44 | 0.17, 1.16 |  |
| **Community-level factors** | | | | | | | | | | | | |
| Governorate |  |  |  |  |  |  |  |  |  |  |  |  |
| Beirut | *1.00* |  | - | *1.00* |  | <0.001 | *1.00* |  | - | *1.00* |  | <0.001 |
| Mount Lebanon | - | - |  | 1.57 | 0.55, 4.48 |  | - | - |  | 1.27 | 0.50, 3.20 |  |
| North | - | - |  | 1.03 | 0.38, 2.78 |  | - | - |  | 1.23 | 0.49, 3.09 |  |
| South and Nabatiyeh | - | - |  | 2.00 | 0.68, 5.89 |  | - | - |  | 2.19 | 0.78, 6.14 |  |
| Bekaa | - | - |  | 4.64 | 1.79, 12.04 |  | - | - |  | 5.74 | 2.39, 13.78 |  |

OR, Odds Ratio; CI, Confidence Interval.

Supplementary Table 3b: Complementary feeding indicators across individual-, household- and community-level factors

|  | Egg and/or flesh food consumption 6-23 months (n=367) | | | Sweet beverage consumption 6-23 months (n=367) | | | Unhealthy food consumption 6-23 months (n=367) | | | No vegetable or fruit consumption 6-23 months (n=367) | | |
| --- | --- | --- | --- | --- | --- | --- | --- | --- | --- | --- | --- | --- |
|  | **OR** | **95% CI** | **p-value** | **OR** | **95% CI** | **p-value** | **OR** | **95% CI** | **p-value** | **OR** | **95% CI** | **p-value** |
| **Individual-level factors** | | | | | | | | | | | | |
| Maternal age (years) |  |  |  |  |  |  |  |  |  |  |  |  |
| 15-24 | *1.00* |  | 0.594 | *1.00* |  | 0.258 | *1.00* |  | 0.040 | *1.00* |  | 0.598 |
| 25-34 | 0.70 | 0.28, 1.78 |  | 0.39 | 0.12, 1.20 |  | 1.12 | 0.49, 2.52 |  | 0.84 | 0.30, 2.31 |  |
| 35-49 | 0.98 | 0.35, 2.75 |  | 0.51 | 0.18, 1.51 |  | 0.51 | 0.19, 1.37 |  | 0.62 | 0.21, 1.82 |  |
| Maternal education level |  |  |  |  |  |  |  |  |  |  |  |  |
| Primary school or less | *1.00* |  | 0.504 | *1.00* |  | 0.023 | *1.00* |  | 0.410 | *1.00* |  | 0.391 |
| Intermediate, high school, or Technical Diploma | 1.72 | 0.60, 4.87 |  | 1.41 | 0.53, 3.73 |  | 0.68 | 0.25, 1.82 |  | 0.53 | 0.20, 1.39 |  |
| University degree or higher | 1.37 | 0.39, 4.74 |  | 0.56 | 0.16, 1.90 |  | 0.48 | 0.16, 1.43 |  | 0.48 | 0.13, 1.73 |  |
| Partner’s education level |  |  |  |  |  |  |  |  |  |  |  |  |
| Primary school or less | *1.00* |  | 0.057 | *1.00* |  | 0.659 | *1.00* |  | 0.207 | *1.00* |  | 0.153 |
| Intermediate, high school, or Technical Diploma | 1.53 | 0.86, 2.74 |  | 0.83 | 0.37, 1.86 |  | 0.69 | 0.34, 1.38 |  | 0.46 | 0.19, 1.10 |  |
| University degree or higher | 2.61 | 1.19, 5.70 |  | 1.25 | 0.45, 3.45 |  | 0.48 | 0.22, 1.08 |  | 0.33 | 0.09, 1.26 |  |
| Maternal working status |  |  |  |  |  |  |  |  |  |  |  |  |
| Working | *1.00* |  | 0.289 | *1.00* |  | 0.376 | *1.00* |  | 0.227 | *1.00* |  | 0.005 |
| Not working | 0.67 | 0.31, 1.42 |  | 1.44 | 0.64, 3.26 |  | 1.69 | 0.72, 4.01 |  | 3.47 | 1.47, 8.19 |  |
| Partner’s working status |  |  |  |  |  |  |  |  |  |  |  |  |
| Working | *1.00* |  | 0.352 | *1.00* |  | 0.387 | *1.00* |  | 0.925 | *1.00* |  | 0.763 |
| Not working | 0.55 | 0.16, 1.95 |  | 0.57 | 0.16, 2.05 |  | 1.06 | 0.32, 3.48 |  | 0.82 | 0.22, 3.03 |  |
| Sex of the child |  |  |  |  |  |  |  |  |  |  |  |  |
| Male | *1.00* |  | 0.255 | *1.00* |  | 0.698 | *1.00* |  | 0.455 | *1.00* |  | 0.245 |
| Female | 1.49 | 0.75, 2.98 |  | 1.09 | 0.70, 1.70 |  | 1.22 | 0.72, 2.07 |  | 0.72 | 0.41, 1.26 |  |
| Child’s age (months) |  |  |  |  |  |  |  |  |  |  |  |  |
| 0–5 | *NA* | *NA* |  | *NA* | *NA* |  | *NA* | *NA* |  | *NA* | *NA* |  |
| 6–11 | *1.00* |  | <0.001 | *1.00* |  | 0.004 | *1.00* |  | <0.001 | *1.00* |  | 0.001 |
| 12–23 | 3.27 | 1.74, 6.13 |  | 2.35 | 1.33, 4.17 |  | 5.36 | 2.46, 11.67 |  | 0.28 | 0.13, 0.61 |  |
| Birth order |  |  |  |  |  |  |  |  |  |  |  |  |
| First-born | *1.00* |  | 0.323 | *1.00* |  | 0.018 | *1.00* |  | 0.491 | *1.00* |  | 0.009 |
| Second to fourth | 1.37 | 0.73, 2.58 |  | 2.18 | 1.25, 3.79 |  | 1.42 | 0.77, 2.61 |  | 0.40 | 0.21, 0.76 |  |
| Fifth or more | 2.30 | 0.77, 6.84 |  | 2.60 | 0.84, 8.03 |  | 1.33 | 0.35, 5.05 |  | 1.29 | 0.42, 4.00 |  |
| Mode of delivery |  |  |  |  |  |  |  |  |  |  |  |  |
| Vaginal delivery | *1.00* |  | 0.710 | *1.00* |  | 0.089 | *1.00* |  | 0.655 | *1.00* |  | 0.817 |
| Cesarean section delivery | 1.12 | 0.61, 2.04 |  | 0.63 | 0.36, 1.08 |  | 0.86 | 0.45, 1.67 |  | 0.89 | 0.33, 2.39 |  |
| Maternal Body Mass Index |  |  |  |  |  |  |  |  |  |  |  |  |
| Normal | *1.00* |  | 0.128 | *1.00* |  | 0.752 | *1.00* |  | 0.427 | *1.00* |  | 0.263 |
| Underweight | 2.47 | 0.21, 29.67 |  | 2.43 | 0.26, 22.78 |  | 1.78 | 0.23, 13.61 |  | 0.85 | 0.12, 5.78 |  |
| Overweight | 0.49 | 0.22, 1.08 |  | 0.97 | 0.47, 1.98 |  | 0.62 | 0.33, 1.17 |  | 1.23 | 0.56, 2.71 |  |
| Obese | 0.43 | 0.18, 1.01 |  | 1.36 | 0.52, 3.60 |  | 0.87 | 0.52, 1.48 |  | 0.46 | 0.17, 1.24 |  |
| Maternal smoking |  |  |  |  |  |  |  |  |  |  |  |  |
| Current/Ex smoker | *1.00* |  | 0.815 | *1.00* |  | 0.128 | *1.00* |  | 0.473 | *1.00* |  | 0.596 |
| Non-smoker | 0.93 | 0.52, 1.67 |  | 0.64 | 0.35, 1.14 |  | 0.82 | 0.47, 1.42 |  | 1.24 | 0.55, 2.79 |  |
| Alcohol drinking |  |  |  |  |  |  |  |  |  |  |  |  |
| Current alcohol drinker | *1.00* |  | 0.733 | *1.00* |  | 0.299 | *1.00* |  | 0.914 | *1.00* |  | 0.056 |
| Not alcohol drinker | 0.87 | 0.38, 1.99 |  | 1.58 | 0.66, 3.76 |  | 1.05 | 0.46, 2.41 |  | 2.80 | 0.97, 8.08 |  |
| Tertiles of maternal knowledge score related to breastfeeding |  |  |  |  |  |  |  |  |  |  |  |  |
| 1 (lowest) | *1.00* |  | 0.988 | *1.00* |  | 0.985 | *1.00* |  | 0.809 | *1.00* |  | 0.769 |
| 2 | 1.01 | 0.50, 2.06 |  | 0.98 | 0.46, 2.12 |  | 0.95 | 0.49, 1.85 |  | 1.39 | 0.47, 4.13 |  |
| 3 (highest) | 1.06 | 0.48, 2.34 |  | 0.95 | 0.50, 1.80 |  | 0.79 | 0.37, 1.68 |  | 1.18 | 0.63, 2.20 |  |
| **Household-level factors** | | | | | | | | | | | | |
| Household monthly income (Lebanese Pounds) |  |  |  |  |  |  |  |  |  |  |  |  |
| <1,000,000 | *1.00* |  | 0.133 | *1.00* |  | 0.055 | *1.00* |  | 0.314 | *1.00* |  | 0.003 |
| 1,000,000-2,000,000 | 2.57 | 1.03, 6.37 |  | 0.59 | 0.25, 1.42 |  | 1.69 | 0.71, 4.05 |  | 0.28 | 0.10, 0.82 |  |
| >2,000,000 | 1.93 | 0.63, 5.89 |  | 0.43 | 0.13, 1.38 |  | 1.04 | 0.41, 2.66 |  | 0.31 | 0.08, 1.15 |  |
| Doesn’t know/Refused to answer | 2.22 | 0.98, 5.03 |  | 1.26 | 0.54, 2.91 |  | 1.86 | 0.82, 4.22 |  | 0.27 | 0.13, 0.58 |  |
| Presence of paid helper in the household |  |  |  |  |  |  |  |  |  |  |  |  |
| No | *1.00* |  | 0.393 | *1.00* |  | 0.414 | *1.00* |  | 0.481 | *1.00* |  | 0.202 |
| Yes | 0.65 | 0.25, 1.74 |  | 0.69 | 0.28, 1.69 |  | 0.68 | 0.24, 1.99 |  | 0.48 | 0.15, 1.49 |  |
| **Community-level factors** | | | | | | | | | | | | |
| Governorate |  |  |  |  |  |  |  |  |  |  |  |  |
| Beirut | *1.00* |  | 0.017 | *1.00* |  | <0.001 | *1.00* |  | 0.321 | *1.00* |  | 0.007 |
| Mount Lebanon | 2.30 | 1.00, 5.28 |  | 0.30 | 0.12, 0.73 |  | 1.27 | 0.56, 2.86 |  | 0.99 | 0.19, 5.12 |  |
| North | 1.02 | 0.42, 2.52 |  | 0.65 | 0.29, 1.49 |  | 1.37 | 0.65, 2.88 |  | 3.20 | 0.62, 16.51 |  |
| South and Nabatiyeh | 2.38 | 0.95, 5.98 |  | 0.47 | 0.15, 1.50 |  | 1.24 | 0.57, 2.68 |  | 0.86 | 0.15, 5.03 |  |
| Bekaa | 3.16 | 1.31, 7.62 |  | 1.19 | 0.54, 2.61 |  | 4.40 | 1.16, 16.76 |  | 0.23 | 0.02, 2.90 |  |

OR, Odds Ratio; CI, Confidence Interval.

Supplementary Table 4: Comparison of the three blocks (levels) of the logistic regression models for the various IYCF considered in the study.

| **Outcome Variable** | **Statistics** | **Block 1 (individual level)** | **Block 2 (household level)** | **Block 3 (community level)** |
| --- | --- | --- | --- | --- |
| **Ever breastfed** | -2Log Likelihood | 300.8^**^ | 278.8^**^ | 275.7 |
|  | Nagelkerke R² | 0.166 | 0.247 | 0.258 |
| **Early initiation of breastfeeding** | -2Log Likelihood | 512.0^*^ | 508.6 | 508.3 |
|  | Nagelkerke R² | 0.072 | 0.082 | 0.083 |
| **Exclusively breastfed for the first two days** | -2Log Likelihood | 539.8^**^ | 528.3^*^ | 527.8 |
|  | Nagelkerke R² | 0.103 | 0.135 | 0.136 |
| **Exclusive breastfeeding under 6 months** | -2Log Likelihood | 89.0 | 87.4 | 87.4 |
|  | Nagelkerke R² | 0.176 | 0.198 | 0.199 |
| **Mixed milk feeding under 6 months** | -2Log Likelihood | 125.4 | 118.9^*^ | 118.9 |
|  | Nagelkerke R² | 0.159 | 0.232 | 0.232 |
| **Continued breastfeeding 12-23 months** | -2Log Likelihood | 116.7^*^ | 113.9 | 106.7 |
|  | Nagelkerke R² | 0.266 | 0.288 | 0.345 |
| **Meeting minimum dietary diversity (6-23 months)** | -2Log Likelihood | 460.5^*^ | 449.7 | 443.9 |
|  | Nagelkerke R² | 0.088 | 0.125 | 0.144 |
| **Meeting minimum meal frequency (6-23 months)** | -2Log Likelihood | 139.7^*^ | 132.0^*^ | 131.2 |
|  | Nagelkerke R² | 0.158 | 0.213 | 0.219 |
| **Meeting minimum acceptable diet (6-23 months)** | -2Log Likelihood | 447.5^*^ | 430.2^*^ | 424.1 |
|  | Nagelkerke R² | 0.085 | 0.145 | 0.165 |
| **Introduction of solid, semi-solid or soft foods between 6-8 months** | Log Likelihood | 30.9 | 29.5 | NA |
|  | Nagelkerke R² | 0.189 | 0.234 | NA |
| **Egg and/or flesh food consumption between 6-23 months** | Log Likelihood | 474.2^**^ | 470.8 | 464.3 |
|  | Nagelkerke R² | 0.120 | 0.131 | 0.152 |
| **Sweet beverage consumption between 6-23 months** | Log Likelihood | 428.2^**^ | 423.5 | 413.8^*^ |
|  | Nagelkerke R² | 0.213 | 0.227 | 0.256 |
| **Unhealthy food consumption between 6-23 months** | Log Likelihood | 455.5^**^ | 451.5^**^ | NA |
|  | Nagelkerke R² | 0.173 | 0.186 | NA |
| **No vegetable or fruit consumption between 6-23 months** | Log Likelihood | 339.9^**^ | 336.1 | 326.4^*^ |
|  | Nagelkerke R² | 0.140 | 0.155 | 0.192 |

^*^p<0.05; ^**^ p<0.001
